# Reduction in disialyl-T antigen levels in mice deficient for both St6galnac3 and St6galnac4 results in blood filling of lymph nodes

**DOI:** 10.1038/s41598-023-37363-y

**Published:** 2023-06-29

**Authors:** Sayaka Fuseya, Hiroyuki Izumi, Ayane Hamano, Yuka Murakami, Riku Suzuki, Rikako Koiwai, Takuto Hayashi, Atsushi Kuno, Satoru Takahashi, Takashi Kudo

**Affiliations:** 1 Laboratory Animal Resource Center in Transborder Medical Research Center, and Department of Anatomy and Embryology, Institute of Medicine, University of Tsukuba, 1-1-1 Tennodai, Tsukuba, Ibaraki 305-8575 Japan; 2 Cellular and Molecular Biotechnology Research Institute, National Institute of Advanced Industrial Science and Technology, Ibaraki, 305-8565 Japan; 3 Graduate School of Comprehensive Human Sciences, University of Tsukuba, Ibaraki, 305-8575 Japan; 4 School of Integrative and Global Majors, University of Tsukuba, Ibaraki, 305-8575 Japan

**Keywords:** Glycobiology, Glycosylation

## Abstract

Sialic acid (SA) is present at the terminal ends of carbohydrate chains in glycoproteins and glycolipids and is involved in various biological phenomena. The biological function of the disialyl-T (SAα2-3Galβ1-3(SAα2-6)GalNAcα1-*O*-Ser/Thr) structure is largely unknown. To elucidate the role of disialyl-T structure and determine the key enzyme from the *N*-acetylgalactosaminide α2,6-sialyltransferase (St6galnac) family involved in its in vivo synthesis, we generated *St6galnac3*- and *St6galnac4*-deficient mice. Both single-knockout mice developed normally without any prominent phenotypic abnormalities. However, the *St6galnac3*::*St6galnact4* double knockout (DKO) mice showed spontaneous hemorrhage of the lymph nodes (LN). To identify the cause of bleeding in the LN, we examined podoplanin, which modifies the disialyl-T structures. The protein expression of podoplanin in the LN of DKO mice was similar to that in wild-type mice. However, the reactivity of MALII lectin, which recognizes disialyl-T, in podoplanin immunoprecipitated from DKO LN was completely abolished. Moreover, the expression of vascular endothelial cadherin was reduced on the cell surface of high endothelial venule (HEV) in the LN, suggesting that hemorrhage was caused by the structural disruption of HEV. These results suggest that podoplanin possesses disialyl-T structure in mice LN and that both St6galnac3 and St6galnac4 are required for disialyl-T synthesis.

## Introduction

**Figure 1:**
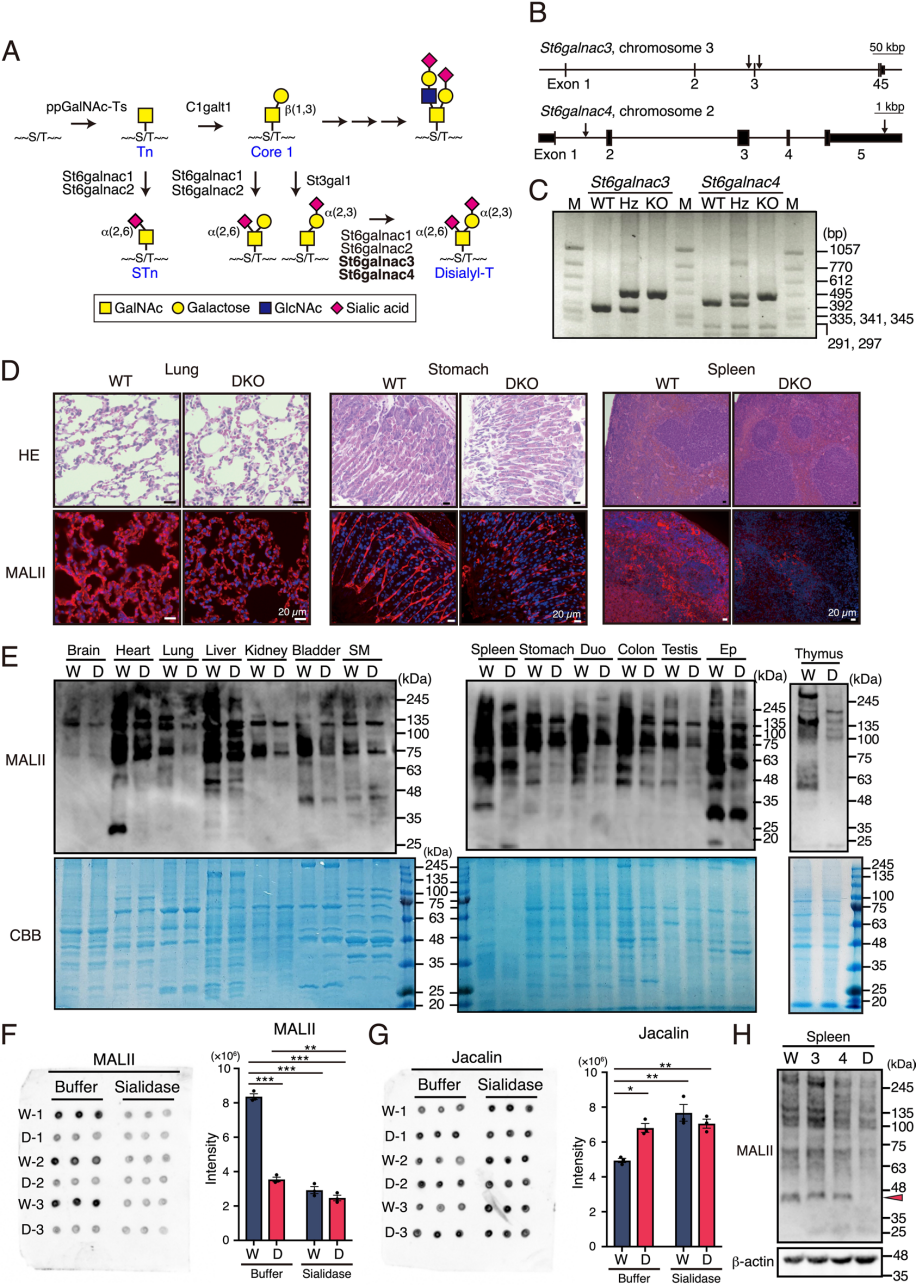
Changes in disialyl-T structure in each tissue. (**A**) Schematic representation of the putative biosynthetic pathway of disialyl-T antigen. Glycosyltransferases involved in biosynthesis are indicated. ppGalNAc-Ts: polypeptide *N*-acetylgalactosaminyltransferases, C1galt1: Core 1 β1,3-galactosyltransferase, St3gal1: β-galactoside α2,3-sialyltransferase 1, St6galnac: GalNAc α2,6-sialyltransferase. (**B**) Genomic structure of the *St6galnac3* and *St6galnac4* genes and the sequences (arrows) targeted by the guide RNAs. (**C**) Genotyping of the *St6galnac3* and *St6galnac4* genes. DNA extracted from the tail of each mouse was used as the template for PCR. WT: wild type, Hz: heterozygous, KO: knockout, M: DNA marker. (**D**) HE and MALII lectin (red) staining using major tissues of WT and DKO mice. The results of other tissues are shown in Fig. S2. (**E**) MALII lectin blot of major tissues from WT and DKO mice. Coomassie brilliant blue-stained acrylamide gels are shown as loading controls. W: WT, D: DKO, SM: Skeletal muscle, Duo: Duodenum, Ep: Epididymis. (**F,G**) Dot blot analysis of brain extracts using MALII (**F**) and Jacalin (**G**) lectin treated with α2–3,6,8,9 Neuraminidase A (sialidase) or buffer as control. Each sample was tested in triplicates (left panel). The intensities of dot blots were measured using ImageJ (right panel). W: WT, 3: 3KO, 4: 4KO, D: DKO. n = 3, Tukey’s multiple comparisons test, * p < 0.05, ** p < 0.01, *** p < 0.001. (**H**) MALII lectin blotting using the spleen extracts of WT, 3KO, 4KO, and DKO mice. Arrowhead: Bands with a significantly reduced signal. W: WT, 3: 3KO, 4: 4KO, D: DKO.

Sialylation is the process of adding sialic acid to the non-reducing ends of various glycoconjugates. Sialic acid is a negatively charged monosaccharide added to many types of glycans, such as *N*-glycans and *O*-glycans (*O*-GalNAc (mucin-type), *O*-Mannose, *O*-Fucose, and *O*-GlcNAc), proteins, and glycolipids, and is involved in many important biological processes such as cell adhesion, signal transduction, and immune regulation^1–3^. Sialylation is tightly regulated by sialyltransferases localized in the Golgi membrane. Twenty different sialyltransferases attach sialic acids to glycans via α2-3, α2-6, and α2-8 linkages. Among them, sialic acid is attached to GalNAc by α2,6-linkages only in mucin-type *O*-glycans and glycolipids. The sialyl-Tn (STn: Sialic acid α2-6GalNAc-Ser/Thr) and disialyl-T (Sialic acid α2-3Galβ1-3(sialic acid α2-6)GalNAc-Ser/Thr) antigens on mucin-type glycans are well-known cancer-associated glycans^4,5^; however, their physiological functions are largely unknown. According to in vitro substrate specificity analysis, the biosynthesis of these antigens is catalyzed by four types of *N*-acetylgalactosaminide α- 2–6-sialyltransferases (St6galnac1, St6galnac2, St6galnac3, and St6galnac4), which attach sialic acid to GalNAc via α2,6-linkages^5,6^ (Fig. 1A). Each pair of *St6galnac1* and *St6galnac2* or *St6galnac3* and *St6galnac4* genes has a high gene sequence homology. In vitro enzymatic analysis has shown that St6galnac1 and St6galnac2 have the highest specific activity for synthesis of the STn antigen, while St6galnac3 and St6galnac4 have the highest specific activity for synthesis of the disialyl-T antigen. STn is almost absent in normal tissues, while disialyl-T is found in normal tissues; however, the roles of St6galnac3 and St6galnac4 and the subsequent disialyl-T antigen synthesized by these enzymes are still unclear.

Podoplanin is an *O*-linked glycosylated type I membrane protein with some highly conserved Platelet Aggregation-stimulating (PLAG) domains. Podoplanin is modified with core 1-derived sialylated* O*-glycans^7–9^. Furthermore, disialyl-T, a type of core 1-derived *O*-glycan, is modified in at least one of its PLAG domains. Podoplanin is expressed in the heart, type 1 alveolar epithelium, renal glomerular podocytes, epithelial cells of the brain choroid plexus, glial cells, lymphatic endothelial cells, fibroblastic reticular cells, and stromal cells in lymph nodes. Podoplanin-knockout (KO) mice exhibit prenatal and postnatal lethality, including cardiac defects, abnormal lung development, lymphedema, hemorrhage of lymphatic vessels, and the absence of the lymph nodes^10–14^. These observations indicate that podoplanin plays important roles in the developmental processes. It is also upregulated in brain tumors, lung cancer, and cervical cancer and is known to be involved in cancer invasion and prognosis^15,16^.

C-type lectin like-receptor 2 (Clec-2) is a functional protein that recognizes podoplanin in vivo. Clec-2 is highly expressed, particularly in platelets and megakaryocytes. The binding of podoplanin to Clec-2 causes platelet activation^17–21^ and plays vital roles in the separation of blood and lymphatic vessels during in vivo development; in maintaining the integrity of high endothelial venule (HEV) structure, a specialized vascular structure in lymph nodes^22^; and in antigen presentation by the dendritic cells. Clec-2 binds through the disialyl-T structure and podoplanin peptide^7,8^. However, the significance of the disialyl-T structure is still unclear, as previous studies have only analyzed *podoplanin-*KO and *Clec-2-*KO cells and mice. Podoplanin may be a promising target protein for developing anti-metastatic drugs, as the Clec-2/podoplanin interaction causes platelet aggregation in the bloodstream, thereby promoting hematogenous tumor metastasis^23,24^. To leverage their interaction for therapy, it is essential to clarify the role of the disialyl-T structure in normal tissue conditions. Therefore, we aimed to elucidate the effect of the disialyl-T structure on podoplanin, determine other additional roles of the disialyl-T structure, and identify the enzyme that synthesizes disialyl-T in vivo. Furthermore, to clarify the role of the disialyl-T structure and determine the enzymes catalyzing the synthesis of this antigen in vivo, we generated double KO mice deficient for *St6galnac3* and *St6galnac4* and for *St6galnac3*::*St6galnac4* and conducted their phenotypic analysis.

## Results

### Generation of St6galnac3 KO, St6galnac4 KO, and St6galnac3::4 DKO mice

In vitro substrate specificity and enzymatic activity analysis in previous studies have demonstrated that four of the six isozymes of the St6galnac family, namely St6galnac1, 2, 3, and 4, are necessary for synthesis of the disialyl-T (SAα2-3Galβ1-3 (SAα2-6) GalNAcα1-*O*-Ser/Thr) structure on mucin-type *O*-glycans (Fig. 1A)^25^. To elucidate the biological functions of the disialyl-T antigen, we mainly focused on St6galnac3 and St6galnac4, which have specific in vitro enzymatic properties, and generated their KO mice using the CRISPR/Cas9 system. Four pX330 vectors containing the target sequences to exclude exon 3 of *St6galnac3* and exons 2–5 of *St6galnac4* were simultaneously microinjected into the pronuclei of fertilized oocytes obtained from C57BL/6 J mice (Fig. 1B). We generated *St6galnac3* KO (3KO), *St6galnac4* KO (4KO), and *St6galnac3*::*St6galnac4* double KO (DKO) mice from a single parent mouse, and the genetically engineered mice were distinguishable upon PCR amplification of the genomic DNA isolated from their tails (Fig. 1C). Mating between *St6galnac3*^+/-^ mice or between *St6galnac4*^+/−^ mice produced offspring with normal Mendelian inheritance (Tables S1 and S2). The DKO mice were generated by crossing *St6galnac3*^+/−^::*St6galnac4*^+/−^ mice with each other.

### Disialyl-T structures are synthesized by St6galnac3 and St6galnac4 in vivo

The 3KO, 4KO, and DKO mice grew to adulthood with no apparent problems and lived for approximately 2 years, similar to the wild-type (WT) mice. There were no changes in the body weights (Fig. S1A) and the number of white blood cells, red blood cells, and platelets (Fig. S1B–D). The weights of major organs of mutant male mice from the four groups were similar to those of the WT mice (Fig. S1E,F).

Lectins are classically used to estimate glycan structures and have been purified from animals and plants; *Maackia Amurensis* Lectin II (MALII, also known as *Maackia amurensis* hemagglutinin; MAH) was reported as a leguminous lectin that recognizes disialyl-T^26^. To confirm changes in the structures of glycans in several tissues, we performed immunofluorescence staining with MALII lectin. MALII reactivity was reduced in the lung, stomach, and spleen in DKO, with no histological changes observed (Fig. 1D). In other tissues, such as the cerebral cortex, cerebellum, heart, duodenum, testis, epididymis, kidney, and epididymal white adipose tissue, hematoxylin and eosin (HE) and MALII staining results were similar in the WT and DKO mice (Fig. S2). Moreover, to compare the amounts of disialyl-T structures in several tissues, we performed lectin blotting using MALII lectin. The results showed a remarkable decrease in the expression of disialyl-T in several tissues, including the brain, heart, lung, liver, kidney, bladder, skeletal muscle, spleen, stomach, duodenum, colon, testis, epididymis, and thymus, in entire lane profile (Fig. 1E).

To examine whether St6galnac3 and St6galnac4 can modify α2,6-sialic acids which constitute disialyl-T, we evaluated the reactivity of MALII and Jacalin by dot blot analysis using brain tissues of WT and DKO mice because expression of the disialyl-T structure has been confirmed in the brain by mass spectrometry^27,28^. Jacalin, a lectin isolated from jackfruit, has binding specificity mainly for galactose-β1,3-*N*-acetylgalactosamine, which constitutes disialyl-T. Hence, Jacalin can be used to evaluate disialyl-T structure as well as the reactivity of MALII, as it does not prefer binding of α2,6-sialylation, while α2,3-sialylation is tolerated^29,30^. Results showed that the reactivity of MALII lectin was reduced in DKO mice compared to that in WT mice, and the difference was eliminated by treatment with α2,3-,6-,8-,9-sialidase from *Arthrobacter ureafaciens* (Fig. 1F). Contrary to these results, the reactivity of Jacalin was increased in DKO mice compared to that in WT mice. Furthermore, the difference in reactivity of Jacalin was also eliminated by sialidase treatment (Fig. 1G). These results suggest reduced reactivity of MALII in DKO mice is due to the loss of α 2,6-sialylation of disialyl-T structure.

In addition, MALII blotting was performed using the spleens of 3KO and 4KO mice. The results indicated that the reduction in MALII reactivity was more significant in DKO mice than in WT, 3KO, and 4KO mice, especially with regard to a band around 45 kDa (Fig. 1H).

To confirm the key enzymes involved in the synthesis of disialyl-T structures in vivo, we evaluated the tissue distribution of the expression of four *St6galnac* genes in 8-week-old male C57BL/6 J mice using quantitative real-time RT-PCR (Fig. S3). Plasmids with known DNA concentrations were used to plot a standard curve to perform a comparative expression analysis of different *St6galnac* genes. The expression of *St6galnac2* and *St6galnac4* was more dominant than that of *St6galnac1* and *St6galnac3* overall. Of the four enzymes, *St6galnac2* showed the highest expression, predominantly in the gastrointestinal and reproductive tissues than in the brain and tissues of immune system including the thymus, spleen, and bone marrow. In contrast, *St6galnac4* showed a higher expression in the brain and immune system than in the gastrointestinal system. In addition, the tissue distribution of *St6galnac3* and *St6galnac4* was similar, and *St6galnac3* and *St6galnac4* were expressed in a wide range of tissues. Lectin blotting (Fig. 1E) and immunofluorescence staining (Figs. 1D, Fig. S2) of MALII lectins showed a reduction in their reactivity in DKO mice compared to WT mice, but did not disappear completely, suggesting that other St6galnac isozymes, such as St6galnac1 and St6galnac2, may be involved in the synthesis of the disialyl-T structure in vivo.

### St6galnac3 and St6galnac4 synthesize the disialyl-T structures expressed in T cells

Because we observed a reduction in the reactivity of MALII in lymphocytes and the spleen of DKO mice (Fig. 1D), the number of T cells in the thymus and T cells/B cells in the spleen was confirmed using immunofluorescence staining and flow cytometry analysis. We observed a decreased reactivity of MALII in CD3-positive T cells of the thymus and spleens of DKO mice (Fig. S4A,B). In contrast, co-localization with MALII was not observed in the B220-positive B cells of the spleens of WT and DKO mice (Fig. S4B). However, flow cytometry analysis of the thymus and spleen showed no significant changes in the populations of major immune cells among all the mutant mice as compared to those in WT mice (Fig. S4C,D).

### St6galnac3::St6galnac4 DKO mice exhibit bleeding in the lymph nodes

**Figure 2:**
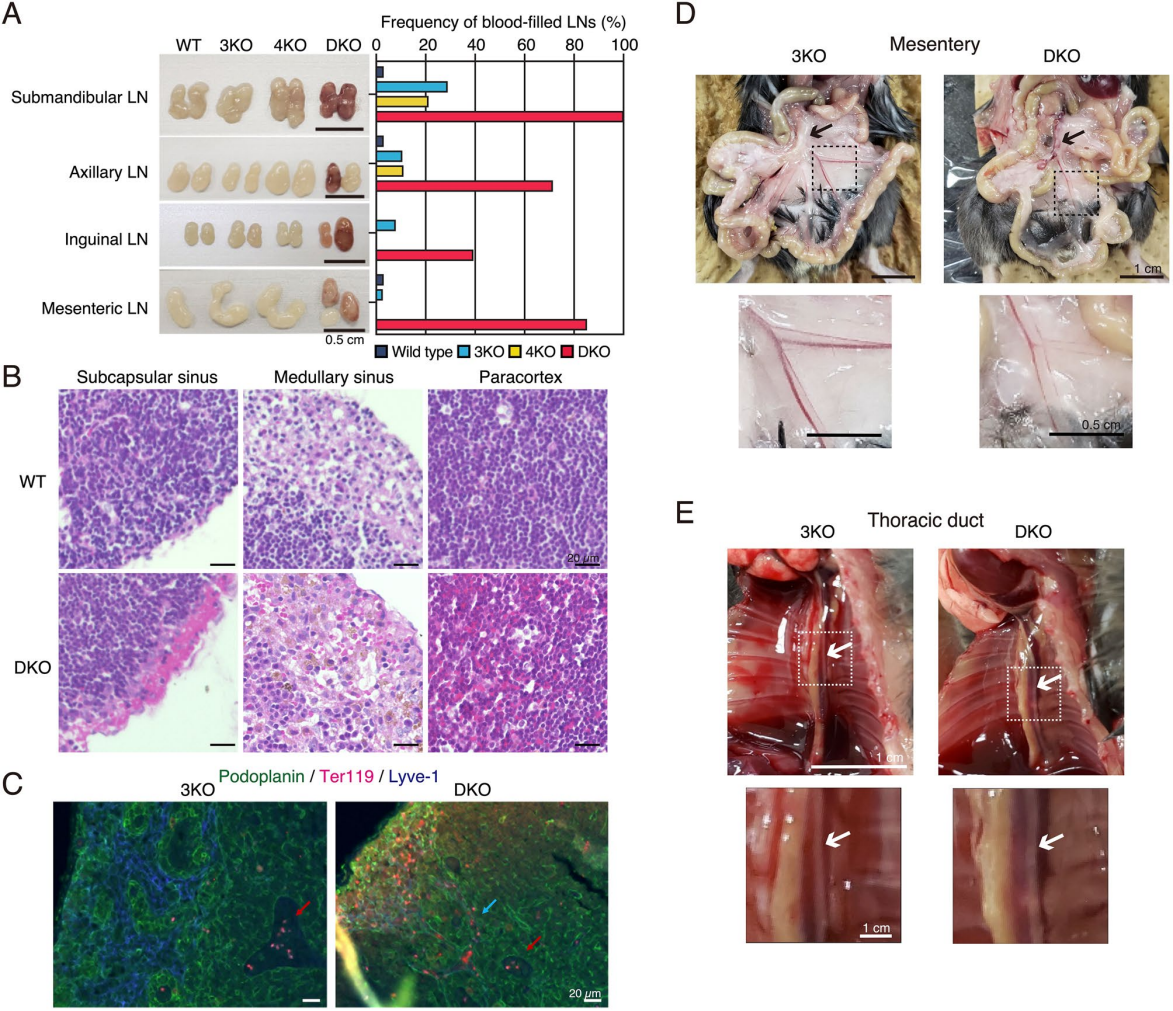
Phenotypes of abnormal lymph nodes in DKO mice. (**A**) Frequency of blood-filled lymph nodes in KO mice. LN: Lymph node. WT: n = 33, 3KO: n = 38, 4KO: n = 19, DKO: n = 28. (**B**) HE staining of the submandibular lymph nodes in DKO mice. Representative pictures of the subcapsular sinus, medullary sinus, and paracortex in LNs. (**C**) Immunostaining results of mesenteric lymph nodes in 3KO (littermate control) and DKO mice. Red arrow: red blood cells within the HEV area. Blue arrow: red blood cells flowed in the lymphatic vessel. Podoplanin (green): a marker for lymphatic endothelial vessels and fibroblastic reticular cells. Ter119 (red): a marker for red blood cells. Lyve-1 (blue): a marker for the lymphatic endothelial vessels. (**D**) Comparison of mesenteric lymph nodes and lymphatic vessels between 3KO and DKO mice. High magnification images (lower panels) of mesenteric lymphatic vessels in 3KO and DKO mice. Lower panels show a magnified image of the inset marked in the upper panels. Arrows indicate the range of mesenteric lymph nodes. (**E**) Comparison of the thoracic duct (white arrows) between 3KO and DKO mice. Lower panels are magnified images of the area marked in the upper panels.

In DKO mice, the peripheral lymph nodes, including submandibular, axillary, inguinal, and mesenteric lymph nodes, showed increased bleeding (Fig. 2A). Particularly, hemorrhages were observed in more than 80% of DKO mice in the submandibular, axillary, and mesenteric lymph nodes. In addition, the submandibular lymph nodes of 3KO and 4KO mice also showed hemorrhages. To investigate the cause of these hemorrhages, we performed histological analysis, using HE staining, of the submandibular lymph nodes. As a result, erythrocytes were identified outside the blood vessels in the subcapsular sinus, medullary sinus, and paracortex in DKO mice (Fig. 2B). Furthermore, to efficiently generate DKO mice, *3KO*:: *St6galnac4*^+/−^ mice were mated with each other and 3KO mice were used as the control and we performed immunostaining for a detailed examination of erythrocyte localization in mesenteric lymph nodes, where the frequency of hemorrhage was particularly high in DKO mice, as shown in Fig. 2A. In 3KO mice, Ter119 signals, which are erythrocyte markers, were not observed in the Lyve-1-positive area that marks the lymphatic vessels (Fig. 2C). However, in DKO mice, Ter119 signals were observed in Lyve-1 and podoplanin which is a marker of lymphatic endothelial cells and fibroblastic reticular cells (Fig. 2C). These results suggest that in DKO mice, erythrocytes leak out of the blood vessels into the lymph nodes and may flow through the lymphatic vessels to the edge of lymph nodes.

During the embryonic stage, the interaction of podoplanin and Clec-2 induces the blood–lymphatic vessel separation^17,19,20,31^. Therefore, we determined whether the development of blood and lymphatic vessels was affected in DKO mice. However, visual inspection revealed no blood inflow into the lymphatic vessels in the mesenteries of DKO mice (Fig. 2D). In addition, blood flow into the thoracic duct was not observed in DKO mice (Fig. 2E), contrary to that in a previous report^32^.

### Mouse podoplanin is modified with disialyl-T structures by St6galnac3 and St6galnac4 in the lymph nodes

**Figure 3:**
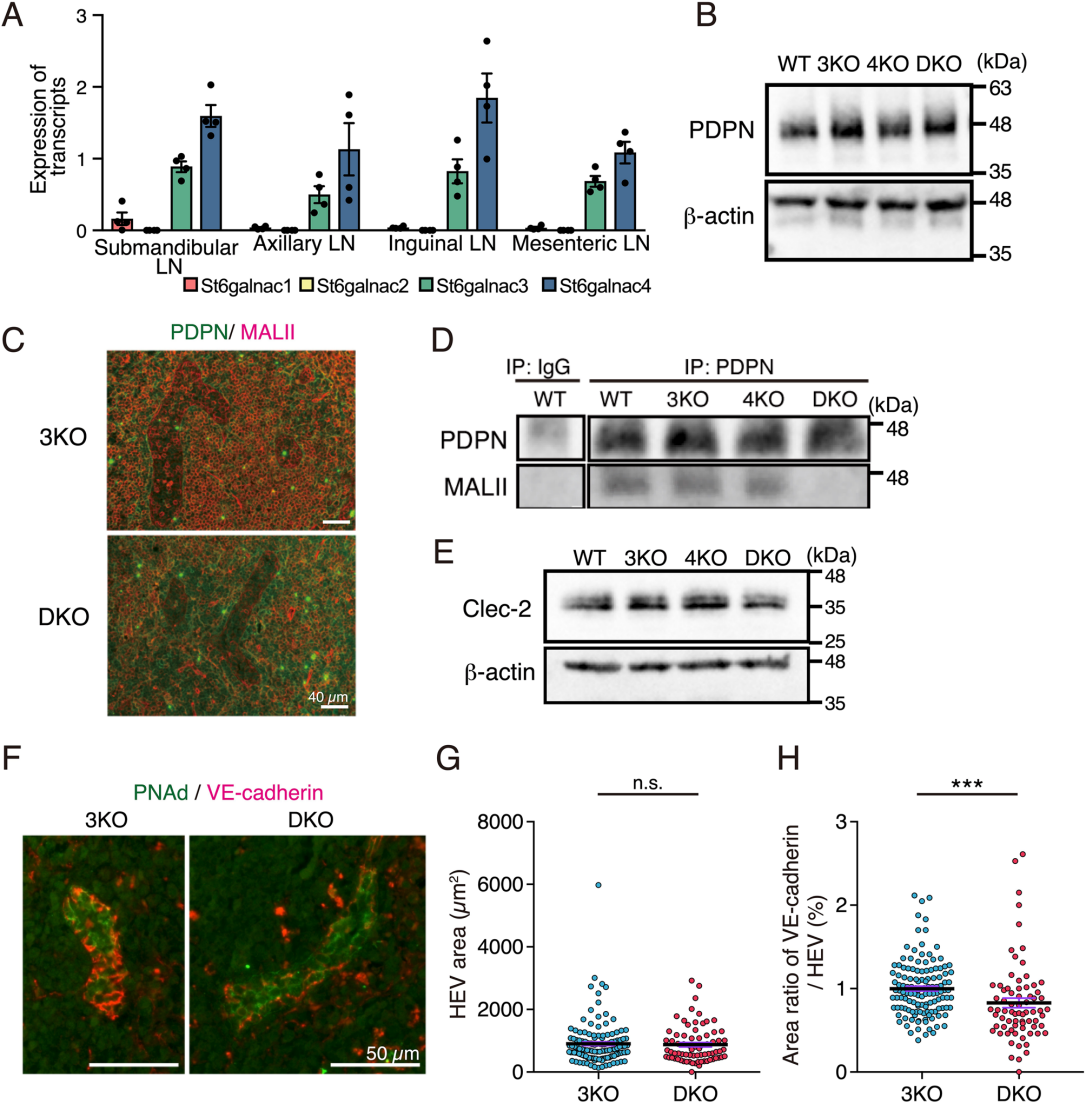
Reduction in disialyl-T structures on podoplanin in the lymph nodes of DKO mice. (**A**) Relative transcript levels of *St6galnac1*, *St6galnac2*, *St6galnac3*, and *St6galnac4* in WT lymph nodes (n = 4). *Hprt* transcript levels were used for normalization. (**B**) Western blotting of podoplanin expression using the lysates of mesenteric lymph nodes in WT, 3KO, 4KO, and DKO mice. (**C**) Immunofluorescence staining of podoplanin (PDPN, green) and MALII lectin (red) in 3KO and DKO mesenteric lymph nodes. (**D**) Western blotting of podoplanin and MALII blotting using immunoprecipitated podoplanin from the lysates of mesenteric lymph nodes in WT, 3KO, 4KO, and DKO mice. Control rat IgG was used for detecting the unspecific bands. The immunoprecipitated samples were detected using an anti-podoplanin antibody and MALII lectin. (**E**) Western blots indicate Clec-2 levels in the lysates of platelets isolated from WT, 3KO, 4KO, and DKO peripheral blood. (**F**) Representative immunofluorescence staining images of VE-cadherin (red) and peripheral node addressin (PNAd, green) in DKO versus littermate control (3KO). (**G,H**) HEV area (**G**) and the area ratio of VE-cadherin/HEV (**H**) quantified using the results of immunofluorescence staining shown in Fig. 3F. HEV area was quantified by measuring the PNAd signal-positive area. 3KO: n = 3 (HEV: n = 271), DKO: n = 3 (HEV: n = 153), two-tailed Mann–Whitney’s rank test, n.s.; not significant, ***p < 0.001. *LN* Lymph node, *IP* immunoprecipitation, *PDPN* podoplanin.

We evaluated the gene expression of 4 *St6galnac* isozymes using quantitative RT-PCR to determine the key enzyme of disialyl-T synthesis in the lymph nodes. *St6galnac3* and *St6galnac4* transcripts levels were dominant in the submandibular, axillary, inguinal, and mesenteric lymph nodes, whereas the expression levels of *St6galnac1* and *St6galnac2* were remarkably low (Fig. 3A).

To confirm whether the phenotype was mediated by the disialyl-T structures modified by the St6galnac3 and St6galnac4, we examined changes in the disialyl-T structures of the mesenteric lymph node extracts.

The expression levels of podoplanin remained unaffected in the lymph node extracts from WT and all KO mice (Fig. 3B). Immunofluorescent staining with MALII lectin showed decreased reactivity of MALII in cells expected to be lymphocytes, such as T cells in the lymph nodes (Fig. 3C). However, the localization of podoplanin and MALII did not overlap. These results were supported by the co-staining of podoplanin and MALII in the kidney (Fig. S5A). Podoplanin is expressed in podocytes and Bowman's capsule epithelial cells of the kidney. MALII only reacted with the glycoproteins expressed in podocytes and not with those expressed in the Bowman's capsule epithelial cells; interestingly, it did not co-localize with podoplanin. This result could either be due to the presence of fewer disialyl-T structures on podoplanin or due to oversaturated signals of MALII reactivity from other cells. Therefore, we performed immunoprecipitation using an anti-podoplanin antibody and determined the reactivity of MALII using the enriched samples (Fig. 3D). MALII signals were detected at similar levels in WT, 3KO, and 4KO mice; however, the signal was lost in DKO mice. Furthermore, we performed immunoprecipitation using an anti-podoplanin antibody to confirm the reactivity of MALII in other tissue extracts. Podoplanin expression levels in the DKO extracts were similar to those in WT extracts from the lung, kidney, and spleen (Fig. S5B). MALII reactivity in the kidney extracts immunoprecipitated with an anti-podoplanin antibody was also lost in the DKO samples compared to that in the WT samples (Fig. S5C). These results indicate that mouse podoplanin is modified with the disialyl-T structure in the lymph nodes and that both St6galnac3 and St6galnac4 are required for its synthesis.

### VE-cadherin is reduced within the HEV area in the lymph nodes of DKO mice

The interaction between podoplanin and Clec-2 plays important roles in the embryonic and adult stages. To investigate whether the hemorrhages of lymph nodes were caused by reduced Clec-2 expression in DKO mice, we performed western blotting using an anti-Clec-2 antibody in the platelet extracts (Fig. 3E). The expression of Clec-2 in all KO mice was found to be similar to that in WT mice.

In the adult stage, the interactions of podoplanin in fibroblastic reticular cells and Clec-2 in platelets contribute to the structural integrity of HEV in the lymph nodes^22^. Therefore, we determined VE-cadherin expression, which is important for the cell–cell adhesion of the endothelial cells in HEV. Immunofluorescence staining using anti-VE-cadherin antibody revealed a decrease in the intensity of VE-cadherin within the HEV region, which is defined as the positive area of the peripheral node addressin (PNAd) signal, in the lymph nodes of DKO mice (Fig. 3F). We performed western blotting using an anti-VE-cadherin (CD144) antibody to compare the expression levels of VE-cadherin in the lymph nodes. VE-cadherin expression remained unchanged in all KO mice compared to that in WT mice (Fig. S6). This may be because VE-cadherin is also expressed in capillary endothelial cells, which masks the reduced expression of VE-cadherin in HEV.

Next, we determined the area of VE-cadherin for each HEV region using the results shown in Fig. 3F. There was no difference in the HEV region between littermate 3KO and DKO mice (Fig. 3G); however, the area of VE-cadherin per HEV region decreased by 20% in DKO mice compared to that in 3KO mice (Fig. 3H). These results suggest that a partial decrease in VE-cadherin expression may promote the leakage of erythrocytes from HEVs.

## Discussion

The role of disialyl-T structures has been largely unknown due to the lack of knowledge regarding the in vivo substrate specificity of the St6galnac family. In this study, we generated 3KO, 4KO, and DKO mice using the CRISPR/Cas9 system and performed phenotypic analysis. MALII lectin analysis revealed that the disialyl-T structure is partially reduced in some tissues of DKO mice. Moreover, we found MALII is required α2,6-sialylation for binding disialyl-T structures by the experiment with sialidase against a report^26^ which α2,6-sialylation in disialyl-T does not seem to be required for MALII binding to disialyl-T.

In this study, we focused on podoplanin, a disialyl-T glycosylated protein. It plays vital roles during developmental and postnatal stages. *Podoplanin* KO mice die after birth due to respiratory failure, with defective differentiation of type I alveolar cells^10^. Furthermore, it has been reported that *Clec-2* KO mice also show abnormal lung development; additionally, the analysis of tissue-specific *podoplanin* KO mice (Tie2-Cre, *podoplanin*^fl/fl^) strongly suggests that the interaction of Clec-2 on platelets with podoplanin on lymphatic endothelial cells of the lung contributes to lung development^33^. *Podoplanin* KO mice also show abnormalities in lymphatic vessels due to the failure of blood–lymphatic vessel separation, resulting in blood-filled lymphatic vessels, edema, and blood–lymphatic discontinuities^11,13,34–36^. The blood–lymphatic vessel-mixed phenotype is also common in mice lacking *C1galt1*, a glycosyltransferase that regulates mucin-type *O*-glycosylation, or inactivated C1galt1^37–39^. Both *podoplanin*- and *Clec-2*-deficient mice also show defects in neurovascular integrity in the embryonic brain^40^. In contrast to our expectations, no abnormalities were observed in the lung or lymphatic vessel development of DKO mice, although reduced reactivity was observed in the lungs of DKO mice using MALII staining. Bleeding abnormalities were observed in the lymph nodes after birth, indicating that DKO mice were healthy during the embryonic stage. These findings indicate that the disialyl-T structures modified by St6galnac3 and St6galnac4 do not regulate the lymphatic system during the embryonic stage but are important for the maintenance of vascular structures in HEV of the lymph nodes during the adult stage. Moreover, the single deletions of St6galnac3 and St6galnac4 did not prevent disialyl-T synthesis in various tissues, suggesting that the other enzyme synthesizes disialyl-T in a compensatory manner.

The function of podoplanin in homeostasis during the postnatal period has been reported based on the analysis of various *podoplanin* conditional KO mice. Blood-filled lymph nodes have also been observed in postnatal lymphatic endothelial cell-specific (Prox1-Cre-ER^T2^) *podoplanin* KO mice^32^. These hemorrhages were attributed to the retrograding of the subclavian vein into the thoracic duct due to the abnormality in the lymphovenous valves. In addition, after birth, the mice with Clec-2-deficient platelets do not show hemorrhage in the lymph nodes, although hemorrhage is observed in their lymph vessels^41^. Therefore, the absence of hemorrhage in the thoracic duct and mesenteric lymphatic vessels in DKO mice suggests that the hemorrhage in lymph nodes is independent of the hemorrhage in lymphatic vessels.

Another Clec-2-podoplanin-mediated mechanism in lymph nodes is the interaction between platelet Clec-2 and podoplanin in fibroblastic reticular cells, which contributes to the integrity of HEVs^22^. According to this report, the platelets activated by podoplanin-Clec-2 interactions induce the expression of Sphingosine-1-phosphate, followed by VE-cadherin in HEVs. Consistent with these findings, we observed a decrease in VE-cadherin within the HEV region in DKO mice. The decrease in VE-cadherin expression was more severe in *podoplanin* KO or *Clec-2* KO than in DKO mice, as was the extent of the hemorrhages. The MALII lectin reactivity of the podoplanin protein purified by immunoprecipitation was lost only in DKO mice compared to that in WT and other single KO mice, suggesting that the disialyl-T structure of podoplanin was largely absent in DKO mice. However, podoplanin and Clec-2 protein levels in DKO mice were similar to those in WT mice. The differences in the extent of hemorrhage and the decrease in VE-cadherin expression may have been a result of alteration in disialyl-T structures. Since Clec-2 recognizes both the amino acids and glycosylation of the podoplanin protein^7,8^, we suspect that the loss of glycosylation may have partially inhibited these interactions in DKO mice, resulting in the impairment of lymph nodes (Fig. S7). The mechanism of lymph node hemorrhage is different from that of lymphatic hemorrhage^41^. Our results are consistent with this finding, as we observed only lymph node hemorrhage in DKO mice, without any signs of lymphatic hemorrhage.

While previous studies have elucidated the function of podoplanin, in the present study, we evaluated the role of glycans in the podoplanin-Clec-2 interaction in vivo by altering the glycan structure of podoplanin. The loss of the disialyl-T structure in podoplanin was observed not only in the lymph nodes but also in the kidney. Hence, podoplanin may function as a signaling molecule rather than just a ligand of Clec-2 in other tissues, even during embryonic development, owing to the abnormalities in these tissues during the embryonic stage, as seen in *podoplanin* KO mice and *Clec-2* KO mice, which were not observed in DKO mice. However, as highlighted in this study, it is important to identify the glycosyltransferases that can synthesize glycan structures in individual organisms, such as mice, and determine the role of these glycans in the cell–cell communication. Disialyl-T, which is expressed in some cancer cells, is also involved in cancer cell invasion and migration^6^. Studies have also reported the role of podoplanin-Clec-2 interactions in cancer metastasis and prognosis^23,24^.

Furthermore, podoplanin derived from glioma cells exacerbates platelet aggregation in tumors in a mouse model^42^. Therefore, targets that inhibit the association between CLEC-2 and podoplanin are suitable candidates for the treatment of anticancer metastasis and anticancer-related thrombosis. However, since podoplanin is also expressed in normal tissues, it is necessary to be aware of the possibility of adverse drug effects. Glycans may be more effective targets for therapy than the proteins themselves, due to their various similar structures and the presence of tissue-specific complementary glycosyltransferases, which may compromise the function of the proteins themselves. In fact, useful some monoclonal antibodies, LpMab, has been reported to specifically recognize abnormally glycosylated podoplanin on cancer cells^43^. This study showed that α 2,6-sialylation of the disialyl-T structures is reduced in vivo by the loss of St6galnac3 and 4. This may be used to artificially induce changes in the disialyl-T structures by genetic modification. Furthermore, with regard to the disialyl-T structure of podoplanin, since only the abnormalities in lymph nodes were noticeable in vivo even after α 2,6-sialylation was reduced, it is possible that the side effects of losing this glycan function may be mild. Our results provide further evidence for targeting this interaction to develop therapeutic strategies to treat cancer.

The disialyl-T structure is a type of core 1-derived *O*-glycan; the incomplete elongation of core 1-derived *O*-glycans during development causes embryonic lethality due to the non-separation of blood and lymphatic vessels. The failure to prevent disialyl-T synthesis by the deletion of St6galnac3 and St6galnac4 is a limitation of this study, which can be addressed by analyzing phenotypic changes in *St6galnac1,2,3,4* quadruple KO mice. We will ultimately investigate the substrate specificity and function of the St6galnac family in vivo, which is not yet clear. Moreover, we believe that these deficient mice would be useful for the evaluation of novel antibodies against cancer and for the assessment of metastasis and malignancy in vivo when cancer is induced.

## Methods

### Animals

Mice were maintained in specific pathogen-free conditions in the Laboratory Animal Resource Center at the University of Tsukuba. All experiments were performed in compliance with the relevant Japanese institutional laws and guidelines as well as approved by the University of Tsukuba Animal Ethics Committee (Authorization Number: 21-173). This experiment complied with the ARRIVE guidelines.

### Generation of *St6galnac3* and *St6galnac4* knockout mice

The *St6galnac3* and *St6galnac4* KO mice were generated using the CRISPR/Cas9 system. In brief, we designed two target sgRNAs, 5′-GAATGTGTACTGCTCGTGCAGGG-3′ and 5′-CCCATCGATTCTGCCTTCTGTTC-3′, against regions located in introns 2 and 3, respectively, of the *St6galnac3* gene of mouse chromosome 3, and two target sgRNAs, 5′-CCCCCAGGTGACCGACCCAGAGG-3′ and 5′-CCCGAATTCCATGTTCCGTTGC-3′, against region located in intron 1 and the untranslated region of exon 5, respectively, of the *St6galnac4* gene of the mouse chromosome 2 (Fig. 1B). Each target sgRNA was cloned into the guide RNA-Cas co-expression plasmid px330-U6-Chimeric_BB-CBh-hSpCas9 vector (Addgene). A mixture of four cloned plasmids was microinjected into the pronuclei of C57BL/6 J fertilized eggs, and living embryos were transferred to pseudopregnant ICR female mice. Genomic DNA Screening was performed using PCR (Fig. 1C); the primers are listed in Table S3.

### Antibodies and lectins

The details regarding antibodies and lectins used for immunofluorescence, flow cytometry, western blotting, immunoprecipitation, and dot blots are provided in Table S4.

### Quantitative analysis of transcripts using RT-qPCR

The total RNA was extracted from various organs of C57BL/6 J and St6galnac-deficient mice using ISOGEN (Nippon Gene, Tokyo, Japan). First-strand cDNA was synthesized using the QuantiTect Reverse Transcription kit (Qiagen, Venlo, Netherland). PCR was performed using reagents from the THUNDERBIRD SYBR qPCR system (Toyobo, Osaka, Japan), and quantification was done using a TP850 Thermal Cycler Dice Real Time System (Takara Bio, Shiga, Japan). The primer sequences used for RT-qPCR are listed in Table S5. Zero blunt TOPO vectors (Thermo Fisher Scientific, Waltham, MA) cloned with DNA fragments of each PCR-amplified *St6galna*c gene were used as templates to create standard curves for absolute quantification of the *St6galnac* genes. The relative levels of transcripts of each *St6galnac* gene were normalized to those of the hypoxanthine guanine phosphoribosyl transferase (*Hprt*) transcripts.

### Analysis of blood indices

Peripheral blood samples from 10- to 18-week-old male mice were obtained from the inferior vena cava and collected in polypropylene tubes containing 100 µl EDTA-2 K. Blood counts were determined using an automated hemocytometer (Nihon Kohden, Tokyo, Japan).

### Flow cytometric analysis

The spleen and thymus were mechanically homogenized and passed through a 70 µm cell strainer. The cells were hemolyzed in ACK Lysing buffer (0.15 M NH_4_Cl, 10 mM KHCO_3_, 1 mM EDTA-2Na) at 23–26 °C for 5 min. Samples were washed three times with 1% Bovine serum albumin (BSA)/ Dulbecco’s phosphate buffered saline (DPBS; Thermo Fisher Scientific); 1 × 10^6^ cells were incubated with each primary antibody against lymphocyte markers in 100 µL 1% BSA/DPBS for 20 min on ice. The cells were then washed twice with 1% BSA/DPBS. To remove dead cells, 4′,6-diamidino-2-phenylindole (DAPI, Dojindo Laboratories, Kumamoto, Japan) was added before flow cytometry. Flow cytometric analysis was performed using CytoFLEX (Beckman Coulter, Brea, CA).

### Histological analysis

Tissues were fixed in Mildform 10 N (Fujifilm Wako, Osaka, Japan) for 16–20 h at 4 °C and then embedded in paraffin. Three micrometer-thick sections were stained with HE after deparaffinization. For immunohistochemical analysis, 3 µm-thick sections were blocked with 5% BSA/phosphate-buffered saline (PBS; 137 mM NaCl, 10 mM Na_2_HPO_4_, 1.8 mM KH_2_PO_4_, 2.7 mM KCl) or a Carbo-free blocking solution (for lectin staining, Vector laboratories, Newark, CA) for 1 h at 23–26 °C after deparaffinization. Next, these sections were incubated with primary antibodies or lectins for 16–20 h at 4 °C. Samples were visualized using Alexa Flour fluorescence-conjugated secondary antibodies (Thermo Fisher Scientific). Nuclei were counterstained with Vibrance Antifade Mounting Medium containing DAPI (Vector laboratories). All sections were observed using a BIOREVO BZ-X800 microscope (Keyence, Osaka, Japan).

VE-cadherin fluorescence intensity was quantified using the hybrid cell count application (Keyence). After the HEV area with positive PNAd signals was manually encircled, the fluorescence intensity of VE-cadherin was automatically quantified in the PNAd-positive area.

### Protein extraction and immunoprecipitation

Tissues were solubilized in lysis buffer (20 mM HEPES (pH 7.4), 1% Triton X-100) containing protease inhibitor cOmplete (Roche, Basel, Switzerland). The lysates were completely solubilized by pipetting or homogenizing using Biomasher II (Nippi, Tokyo, Japan), if necessary. Protein quantification was performed using the DC Protein Assay Kit (Bio-rad, Hercules, CA) and a microplate reader (Bio-Rad).

Total protein lysate (100 µg) was incubated with 10–20 µL Protein A/G PLUS-Agarose (Santa Cruz, Dallas, TX, sc-2003) at 4 °C for 2 h to reduce non-specific binding. Then, the samples were incubated with anti-podoplanin or control anti-rat immunoglobulin G antibodies for 16–20 h at 4 °C and immunoprecipitated using Protein A/G PLUS-Agarose at 4 °C for 1 h. The reaction solution was washed three times with lysis buffer containing a protein inhibitor. The solution was reduced using SDS sample buffer at 95 °C for 5 min.

### Western blotting and lectin blotting

The tissue extracts were separated on 7.5% or 10% SDS–polyacrylamide gels using the ERICA XV PANTERA SYSTEM (DRC, Osaka, Japan) and then transferred to Immobilon-P membrane (MilliporeSigma, Burlington, MA) using a wet blotting system (Nihon Eido, Tokyo, Japan). After blocking with 5% skim milk/PBS containing 0.1% Tween 20 (PBS-T) or a Carbo-free blocking solution containing 0.1% Tween 20 for 1 h at 23–26 °C, the membrane was incubated with primary antibodies/1% skim milk/PBS-T or lectins/blocking buffer for 16–20 h at 4 °C. The membrane was washed three times with PBS-T for 5 min and further incubated with secondary antibodies for 1 h at 23–26 °C. After washing three times with PBS-T, immunoblots were developed using Immobilon western chemiluminescent horseradish peroxidase substrate (MilliporeSigma) and visualized in the iBright imaging system (Thermo Fisher Scientific).

### Dot blot analysis

The brain extracts were incubated with α2-3,6,8,9 Neuraminidase A (New England labs, Ipswich, MA, P0722S) derived from *Arthrobacter ureafaciens* or reaction buffer for 1 h at 37 °C. The treated extracts were dotted onto the Amersham Hybond ECL (Cytiva, Tokyo, Japan, RPN82D). The membrane was dried for 30 min at 23–26 °C and then wetted with PBS-T. After blocking with a Carbo-free blocking solution containing 0.1% Tween 20 for 1 h at 23–26 °C, the membrane was incubated with each lectin/blocking buffer for 16–20 h at 4 °C. The membrane was washed three times with PBS-T for 5 min and then incubated with streptavidin-HRP/blocking buffer for 1 h at 23–26 °C. After washing three times with PBS-T for 5 min, immunoblots were developed using the Immobilon western chemiluminescent horseradish peroxidase substrate (MilliporeSigma) and visualized in the iBright imaging system (Thermo Fisher Scientific). The intensity of each dot was quantified by the plugin tool Band/Peak Quantification Tool^44^ of ImageJ (version 1.53a, Wayne Rasband, NIH, USA).

### Statistical analysis

Statistical analyses were performed using Prism 7 (version 7.0e, GraphPad Software, San Diego, CA), with statistical significance set at 5% (p < 0.05). Data variation shown in the figures is represented as the standard error of the mean (SEM).

## Supplementary Information

Supplementary Information.

## Data Availability

All data are included in this manuscript and the supplemental information. Original western blot images are presented in Supplemental Figs. S8–S11. All other data are available upon reasonable request from the corresponding author(s).

## References

[1] Christiansen MN (2014). Cell surface protein glycosylation in cancer. Proteomics.

[2] Pearce OM, Laubli H (2016). Sialic acids in cancer biology and immunity. Glycobiology.

[3] Varki A (2017). Biological roles of glycans. Glycobiology.

[4] Kudelka MR, Ju T, Heimburg-Molinaro J, Cummings RD (2015). Simple sugars to complex disease–mucin-type O-glycans in cancer. Adv. Cancer Res..

[5] Hugonnet M, Singh P, Haas Q, von Gunten S (2012). The distinct roles of sialyltransferases in cancer biology and onco-immunology. Front. Immunol..

[6] Dimitroff CJ (2015). Galectin-binding O-glycosylations as regulators of malignancy. Cancer Res..

[7] Kato Y (2008). Molecular analysis of the pathophysiological binding of the platelet aggregation-inducing factor podoplanin to the C-type lectin-like receptor CLEC-2. Cancer Sci..

[8] Nagae M (2014). A platform of C-type lectin-like receptor CLEC-2 for binding O-glycosylated podoplanin and nonglycosylated rhodocytin. Structure.

[9] Fuseya S (2020). Mice lacking core 1-derived O-glycan in podocytes develop transient proteinuria, resulting in focal segmental glomerulosclerosis. Biochem. Biophys. Res. Commun..

[10] Ramirez MI (2003). T1alpha, a lung type I cell differentiation gene, is required for normal lung cell proliferation and alveolus formation at birth. Dev. Biol..

[11] Schacht V (2003). T1alpha/podoplanin deficiency disrupts normal lymphatic vasculature formation and causes lymphedema. EMBO J..

[12] Mahtab EA (2008). Cardiac malformations and myocardial abnormalities in podoplanin knockout mouse embryos: Correlation with abnormal epicardial development. Dev. Dyn..

[13] Uhrin P (2010). Novel function for blood platelets and podoplanin in developmental separation of blood and lymphatic circulation. Blood.

[14] Peters A (2011). Th17 cells induce ectopic lymphoid follicles in central nervous system tissue inflammation. Immunity.

[15] Krishnan H (2018). Podoplanin: An emerging cancer biomarker and therapeutic target. Cancer Sci..

[16] Quintanilla M, Montero-Montero L, Renart J, Martin-Villar E (2019). Podoplanin in inflammation and cancer. Int. J. Mol. Sci..

[17] Suzuki-Inoue K (2010). Essential in vivo roles of the C-type lectin receptor CLEC-2: Embryonic/neonatal lethality of CLEC-2-deficient mice by blood/lymphatic misconnections and impaired thrombus formation of CLEC-2-deficient platelets. J. Biol. Chem..

[18] Acton SE (2012). Podoplanin-rich stromal networks induce dendritic cell motility via activation of the C-type lectin receptor CLEC-2. Immunity.

[19] Hess PR (2014). Platelets mediate lymphovenous hemostasis to maintain blood-lymphatic separation throughout life. J. Clin. Invest..

[20] Benezech C (2014). CLEC-2 is required for development and maintenance of lymph nodes. Blood.

[21] Nakamura-Ishizu A, Takubo K, Kobayashi H, Suzuki-Inoue K, Suda T (2015). CLEC-2 in megakaryocytes is critical for maintenance of hematopoietic stem cells in the bone marrow. J. Exp. Med..

[22] Herzog BH (2013). Podoplanin maintains high endothelial venule integrity by interacting with platelet CLEC-2. Nature.

[23] Shirai T (2017). C-type lectin-like receptor 2 promotes hematogenous tumor metastasis and prothrombotic state in tumor-bearing mice. J. Thromb. Haemost..

[24] Suzuki-Inoue K (2018). Roles of the CLEC-2-podoplanin interaction in tumor progression. Platelets.

[25] Kurosawa N, Kojima N, Inoue M, Hamamoto T, Tsuji S (1994). Cloning and expression of Gal beta 1,3GalNAc-specific GalNAc alpha 2,6-sialyltransferase. J. Biol. Chem..

[26] Geisler C, Jarvis DL (2011). Effective glycoanalysis with Maackia amurensis lectins requires a clear understanding of their binding specificities. Glycobiology.

[27] Ohmi Y (2021). Majority of alpha2,6-sialylated glycans in the adult mouse brain exist in O-glycans: SALSA-MS analysis for knockout mice of alpha2,6-sialyltransferase genes. Glycobiology.

[28] Williams SE (2022). Mammalian brain glycoproteins exhibit diminished glycan complexity compared to other tissues. Nat. Commun..

[29] Kuno A (2009). Focused differential glycan analysis with the platform antibody-assisted lectin profiling for glycan-related biomarker verification. Mol. Cell Proteomics.

[30] Takeshita M (2016). Alteration of matrix metalloproteinase-3 O-glycan structure as a biomarker for disease activity of rheumatoid arthritis. Arthritis Res. Ther..

[31] Osada M (2012). Platelet activation receptor CLEC-2 regulates blood/lymphatic vessel separation by inhibiting proliferation, migration, and tube formation of lymphatic endothelial cells. J. Biol. Chem..

[32] Bianchi R (2017). Postnatal deletion of podoplanin in lymphatic endothelium results in blood filling of the lymphatic system and impairs dendritic cell migration to lymph nodes. Arterioscler Thromb. Vasc. Biol..

[33] Tsukiji N (2018). Platelets play an essential role in murine lung development through Clec-2/podoplanin interaction. Blood.

[34] Navarro-Nunez L, Langan SA, Nash GB, Watson SP (2013). The physiological and pathophysiological roles of platelet CLEC-2. Thromb. Haemost..

[35] Pan Y, Xia L (2015). Emerging roles of podoplanin in vascular development and homeostasis. Front. Med..

[36] Suzuki-Inoue K, Osada M, Ozaki Y (2017). Physiologic and pathophysiologic roles of interaction between C-type lectin-like receptor 2 and podoplanin: Partners from in utero to adulthood. J. Thromb. Haemost..

[37] Xia L (2004). Defective angiogenesis and fatal embryonic hemorrhage in mice lacking core 1-derived O-glycans. J. Cell Biol..

[38] Fu J (2008). Endothelial cell O-glycan deficiency causes blood/lymphatic misconnections and consequent fatty liver disease in mice. J. Clin. Invest..

[39] Wang Y (2010). Cosmc is an essential chaperone for correct protein O-glycosylation. Proc. Natl. Acad. Sci. U. S. A..

[40] Lowe KL (2015). Podoplanin and CLEC-2 drive cerebrovascular patterning and integrity during development. Blood.

[41] Haining EJ (2021). Lymphatic blood filling in CLEC-2-deficient mouse models. Platelets.

[42] Costa B (2019). Intratumoral platelet aggregate formation in a murine preclinical glioma model depends on podoplanin expression on tumor cells. Blood Adv..

[43] Yamada S (2017). LpMab-23: A cancer-specific monoclonal antibody against human podoplanin. Monoclon. Antib. Immunodiagn. Immunother..

[44] Ohgane, K. & Yoshioka, H. Quantification of gel bands by an Image J macro, band/peak quantification tool. *Protocols.io* (2019). 10.17504/protocols.io.7vghn3w

