# Supplementary material for: Reduction in disialyl-T antigen levels in mice deficient for both St6galnac3 and St6galnac4 results in blood filling of lymph nodes
Source: Sci Rep. 2023 Jun 29;13:10582. doi: 10.1038/s41598-023-37363-y (PMC10310836; doi:10.1038/s41598-023-37363-y)
Supplement: Supplementary file 1 — Supplementary Information. [file 41598_2023_37363_MOESM1_ESM.pdf]

Reduction in disialyl-T antigen levels in mice deficient for both St6galnac3 and St6galnac4 results in blood filling of lymphatic nodes

Sayaka Fuseya[1,2], Hiroyuki Izumi[1,3], Ayane Hamano[1], Yuka Murakami[1,4], Riku Suzuki[1], Rikako Koiwai[1], Takuto Hayashi[1], Atsushi Kuno[2], Satoru Takahashi[1*], Takashi Kudo[1*].

[1] Laboratory Animal Resource Center in Transborder Medical Research Center, and Department of Anatomy and Embryology, Institute of Medicine, University of Tsukuba, Ibaraki 305-8575, Japan
[2] Cellular and Molecular Biotechnology Research Institute, National Institute of Advanced Industrial Science and Technology, Ibaraki 305-8565, Japan
[3] Master’ s Program in Medical Sciences, Graduate School of Comprehensive Human Sciences, University of Tsukuba, Ibaraki 305-8575, Japan
[4] Ph.D. Program in Human Biology, School of Integrative and Global Majors, University of Tsukuba, Ibaraki 305-8575, Japan

Table S1. Genotypic analysis of offspring from *St6galnac3* heterozygous intercrosses.

	+/+	+/-	-/-
Male (n)	42	105	36
Female (n)	38	80	43
Total (n)	80	185	79
Total (%)	23	54	23

Table S2. Genotypic analysis of offspring from *St6galnac4* heterozygous intercrosses.

	+/+	+/-	-/-
Male (n)	41	82	36
Female (n)	44	90	40
Total (n)	85	172	76
Total (%)	25	52	23

Table S3. Primer sequences for genotyping PCR and PCR product size.

PCR	Primer	Sequence	Size (bp)
1	St6galnac3 Genotype RF	5′-TGGAAAGGACAGGTGAGTCC-3′	St6galnac3 WT: 373
	St6galnac3 Genotype RR	5′-AGGAGCCACAATTTTCATGC-3′	
2	St6galnac3 Genotype LF	5′-TGGATGACCCTCTTCTCCAG-3′	St6galnac3 KO: 484
	St6galnac3 Genotype RR	5′-AGGAGCCACAATTTTCATGC-3′	
3	St6galnac4 Genotype RF	5′-TAGGTGGCATCAGTGCAAAA-3′	St6galnac4 WT: 439
	St6galnac4 Genotype RR	5′-GCCAGCTCTCTACCATGAGC-3′	
4	St6galnac4 Genotype LF	5′-GGGCTGTTCTACACCTCTGC-3′	St6galnac4 KO: 485
	St6galnac4 Genotype RR	5′-GCCAGCTCTCTACCATGAGC-3′	

Table S4. Antibodies and lectins used for analyses.

Name	Clone	Company	Catalog No.	Dilution
Maackia Amurensis Lectin II (MAL II), Biotinylated	-	Vector laboratories	B-1265	(IF) 1:100<br>(WB) 1:500<br>(Dot) 1:100
Artocarpus integrifolia (jackfruit) seed (Jacalin), Biotinylated	-	Vector laboratories	B-1155-5	(Dot) 1:500
PE anti-mouse CD3ε Antibody	145-2C11	BioLegend®	100308	(FC) 1:100
FITC Rat Anti-Mouse CD4	RM4-5	BD Biosciences	553046	(FC) 1:100
PerCP anti-mouse CD8a Antibody	53-6.7	BioLegend®	100732	(FC) 1:100
CD45R (B220) Monoclonal Antibody (RA3-6B2), APC	RA3-6B2	Invitrogen™, eBioscience™	17-0452-82	(FC) 1:100
APC/ Cyanine7 anti-mouse CD19 Antibody	6D5	BioLegend®	115529	(FC) 1:100
PE/Cyanine7 anti-mouse/human CD11b Antibody	M1/70	BioLegend®	101216	(FC) 1:100
Brilliant Violet 510 anti-mouse CD11c Antibody	N418	BioLegend®	117338	(FC) 1:100
Alexa Fluor 488 anti-mouse CD3 Antibody	17A2	BioLegend®	100212	(IF) 1:100
PE anti-mouse/human CD45R/B220 Antibody	RA3-6B2	BioLegend®	103208	(IF) 1:50
Anti-Mouse Podoplanin Antibody	-	R&D Systems	AF3244	(IF) 1:20<br>(WB) 1:1,000
Anti-Aggrus (Podoplanin) (Mouse) mAb	8F11	Medical & Biological Laboratories Co., LTD.	D190-3	(IP) 1:150
Rat IgG2a (isotype control)	2H3	Medical & Biological Laboratories Co., LTD.	M081-3	(IP) 1:150
PE anti-mouse Ter-119 /Erythroid cells Antibody	TER-119	BioLegend®	116207	(IF) 1:50
LYVE1 Monoclonal Antibody (ALY7), Biotin	ALY7	Invitrogen™, eBioscience™	13-0443-82	(IF) 1:50
Human/Mouse/Rat CLEC-2/CLEC1B Antibody	-	R&D Systems	AF1718	(WB) 1:200
Purified anti-mouse/human PNAd Antibody	MECA-79	BioLegend®	120802	(IF) 1:100
CD144 (VE-cadherin) Monoclonal Antibody	eBioBV13 (BV13)	Invitrogen™, eBioscience™	14-1441-82	(WB) 1:1,000
Purified anti-mouse CD144 (VE-cadherin) Antibody	VECD1	BioLegend®	138101	(IF) 1:100
Biotin anti-rat IgM Antibody	MRM-47	BioLegend®	408903	(IF) 1:100
Donkey anti-Goat IgG (H+L) Cross-Adsorbed Secondary Antibody, Alexa Fluor™ 488	-	Invitrogen™	A11055	(IF) 1:2,000
Goat anti-Rat IgG (H+L) Cross-Adsorbed Secondary Antibody, Alexa Fluor™ 594	-	Invitrogen™	A11007	(IF) 1:2,000
Streptavidin, Alexa Fluor™ 488 conjugate	-	Invitrogen™	S11223	(IF) 1:2,000
Streptavidin, Alexa Flour™ 350 conjugate	-	Invitrogen™	S11249	(IF) 1:2,000
Streptavidin, Alexa Flour™ 546 conjugate	-	Invitrogen™	S11225	(IF) 1:2,000
Streptavidin (HRP)	-	Abcam	Ab7403	(WB) 1:5,000<br>(Dot) 1:5,000
Rabbit anti-Goat IgG (H+L) Secondary Antibody, HRP	-	ZYMED	81-1620	(WB) 1:5,000
Goat Anti-Rat IgG H&L (HRP)	-	Abcam	Ab97057	(WB) 1:5,000
Anti-β-actin pAb-HRP-DirecT	-	Medical & Biological Laboratories Co., LTD.	PM053-7	(WB) 1:5,000

* IF: Immunofluorescence, WB: Western blotting, FC: Flow cytometry, IP: Immunoprecipitation, Dot: Dot blot

Table S5. Primer sequences for RT-qPCR.

PCR	Primer	Sequence
1	St6galnac1 Fw	5′-GTACTCCAAGTGTATCACCTGTG-3′
	St6galnac1 Rv	5′-GTCGGAAAACATAGTCATGGCT-3′
2	St6galnac2 Fw	5′-TCCCACGAAGTCATTGCCTC-3′
	St6galnac2 Rv	5′-GGCACAGCGAATACAGCTTAG-3′
3	St6galnac3 Fw	5′-CTTTGCCCTACACATTCAGGC-3′
	St6galnac3 Rv	5′-CTGCCCGACCATTTGACCT-3′
4	St6galnac4 Fw	5′-CACTGAACGCATGATGGCCTA-3′
	St6galnac4 Rv	5′-GTAGACCACGATCTCCTCGC-3′

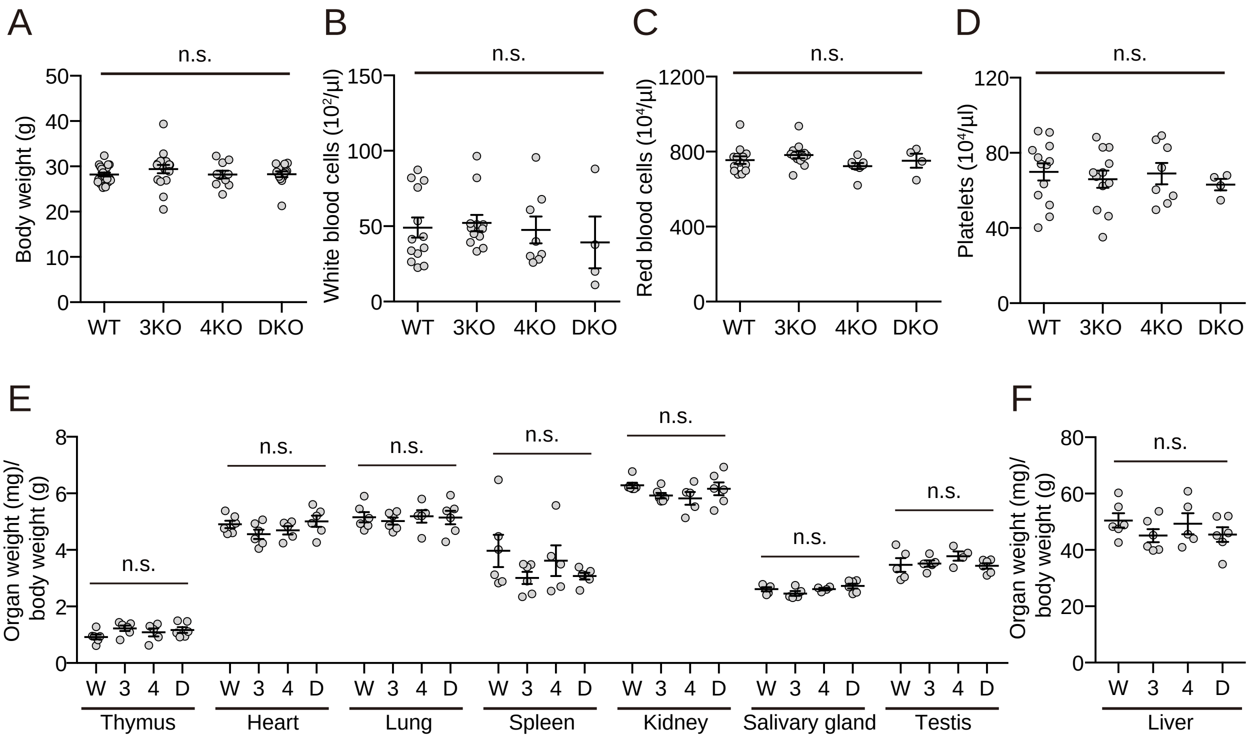

Figure S1. The body and organ weights and blood parameters of WT, 3KO, 4KO, and DKO mice. (A) Body weights of 13- to 14-week-old WT (n = 21), 3KO (n = 18), 4KO (n = 10), and DKO (n = 14) male mice. Comparison with WT using Dunnett's test, n.s.: not significant. (B, C, D) Counts of white blood cells (B), red blood cells (C), and platelets (D) in peripheral blood obtained from the inferior vena cava in 10- to 18-week-old WT (n = 13), 3KO (n = 12), 4KO (n = 8), and DKO (n = 4) male mice. Comparison with WT using Dunnett's test, n.s.: not significant. (E, F) Weights of major organs in 9- to 16-week-old male mice. These data were normalized by body weight. n = 6. Comparison with WT using Dunnett's test, n.s.: not significant.

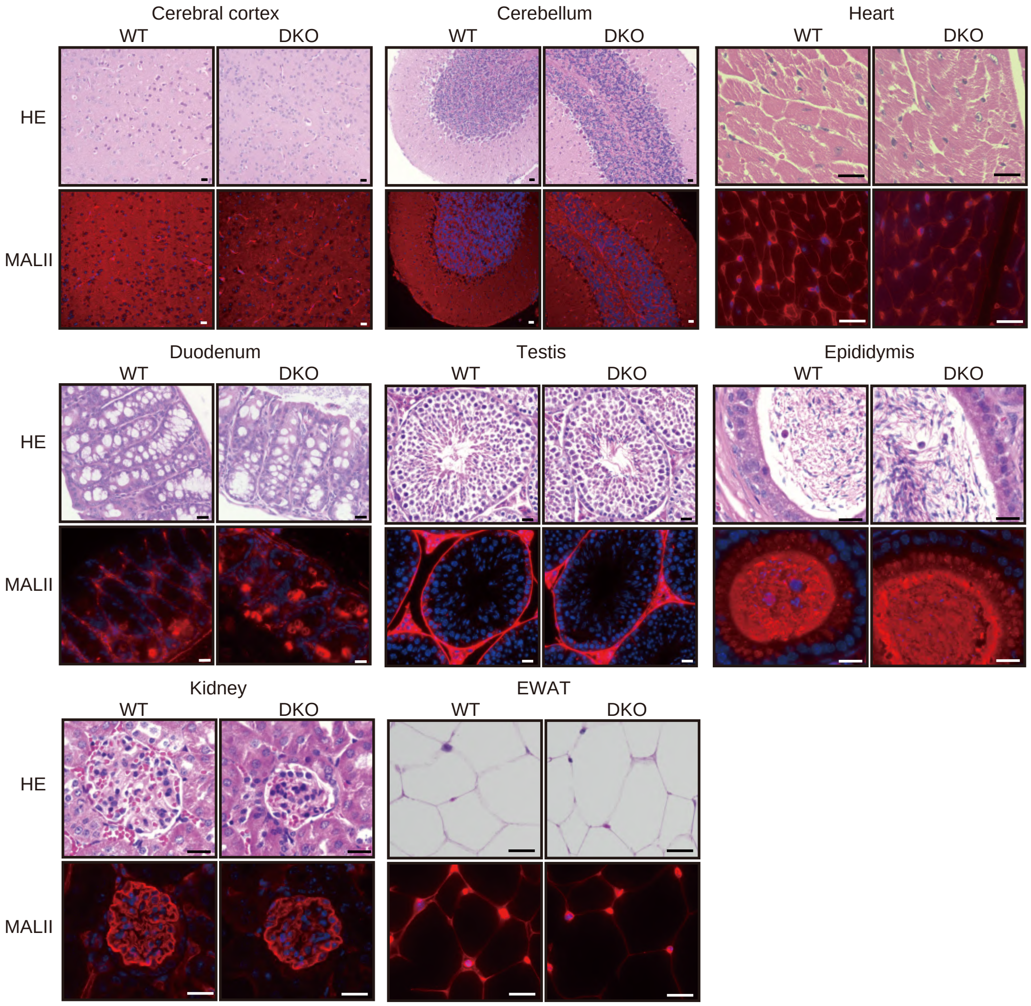

Figure S2. Hematoxylin and eosin (HE) and MALII lectin staining of the cerebral cortex, cerebellum, heart, duodenum, testis, epididymis, kidney, and epididymal white adipose tissue (EWAT) in WT and DKO. Scale bar; 20 μm.

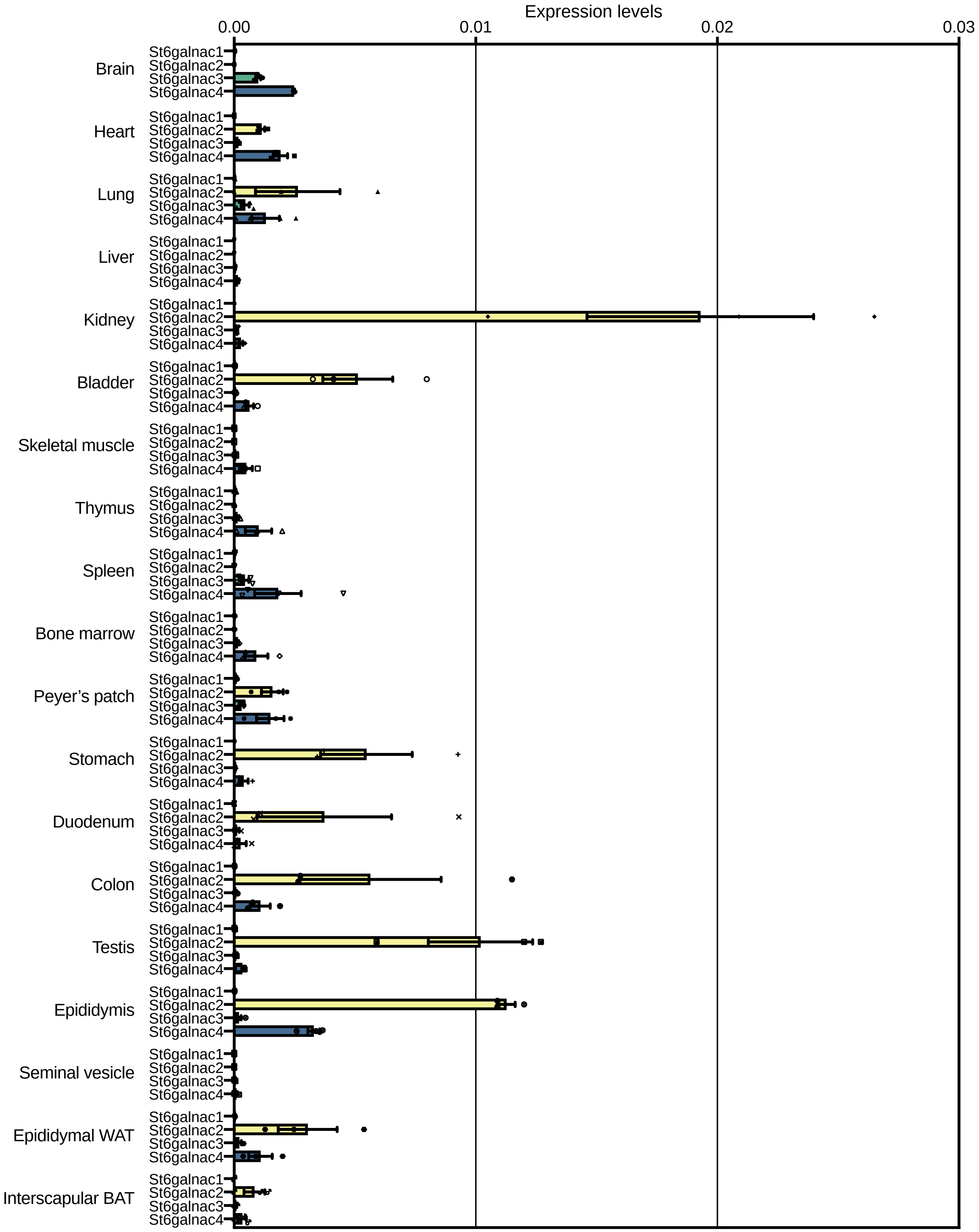

Figure S3. Tissue distribution of four St6galnac transcripts in C57BL/6J male mice (8 weeks old) analyzed using RT-qPCR. WAT: white adipose tissue, BAT: brown adipose tissue. Brain, Heart, Liver, Bladder, Skeletal muscle, Thymus, Bone marrow, Payer’ s Patch, Stomach, Duodenum, Colon, Testis, Seminal vesicle, Epididymal WAT, Interscapular BAT: n = 3, Lung, Kidney, Spleen, Epididymis: n = 3-4.

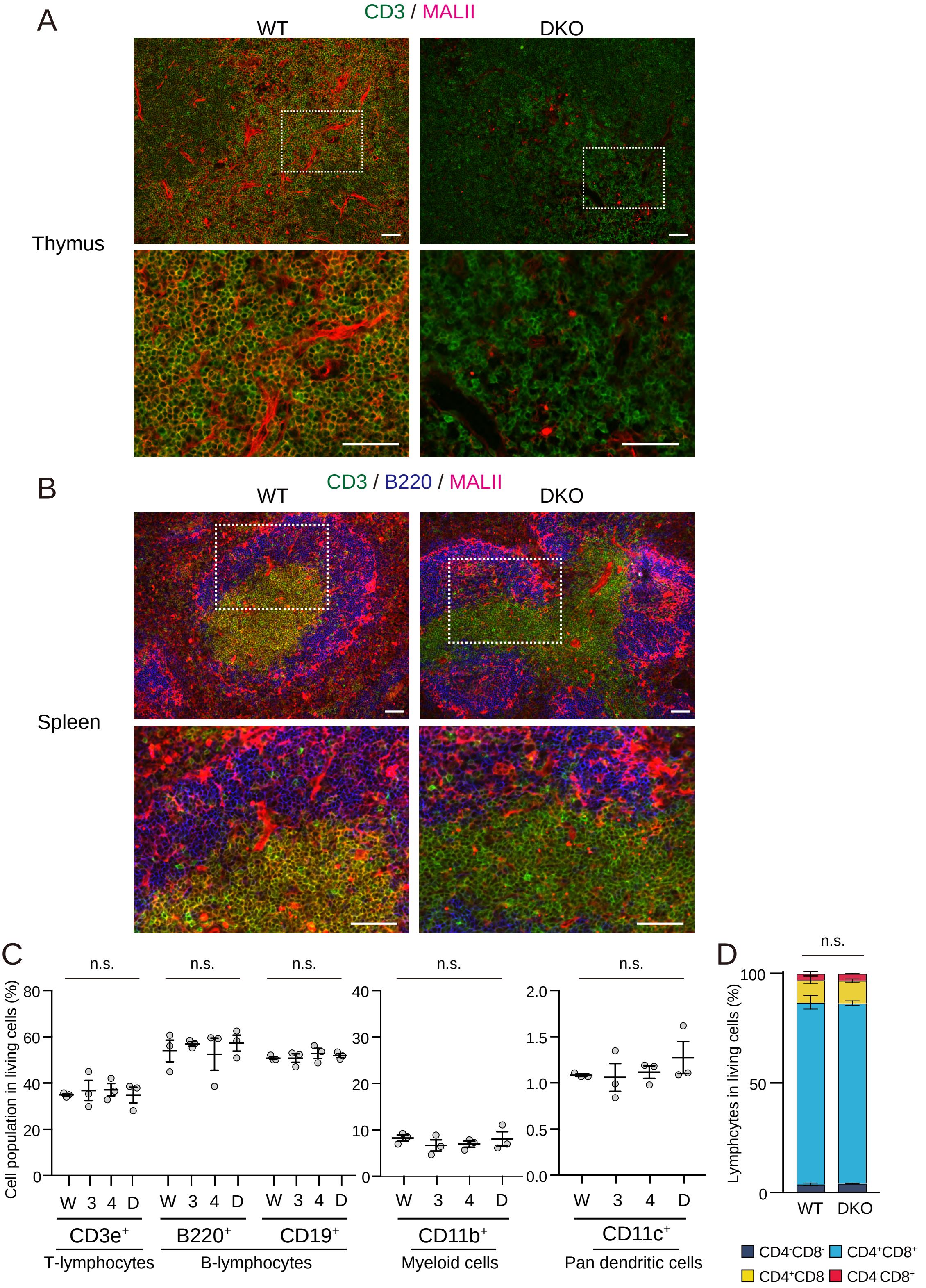

Figure S4. Phenotype of immune cells in DKO mice.
(A) Immunofluorescence staining of CD3 (green) and MALII lectin (red) in the thymus. Scale bar; 50 µm. (B) Immunofluorescence staining of CD3 (green), B220 (blue), and MALII lectin (red) in the spleen. Scale bar; 50 µm. (C) Cell populations of the immune cell lineage in the spleen of each KO mouse (n = 3). Comparison with WT using Dunnett's test, n.s.: not significant, mean ± SEM. (D) Stacked bar graph of CD4 and CD8 lineage T cells in the thymus of WT and DKO mice (n = 3). Bonferroni's multiple comparison test, n.s.; not significant.

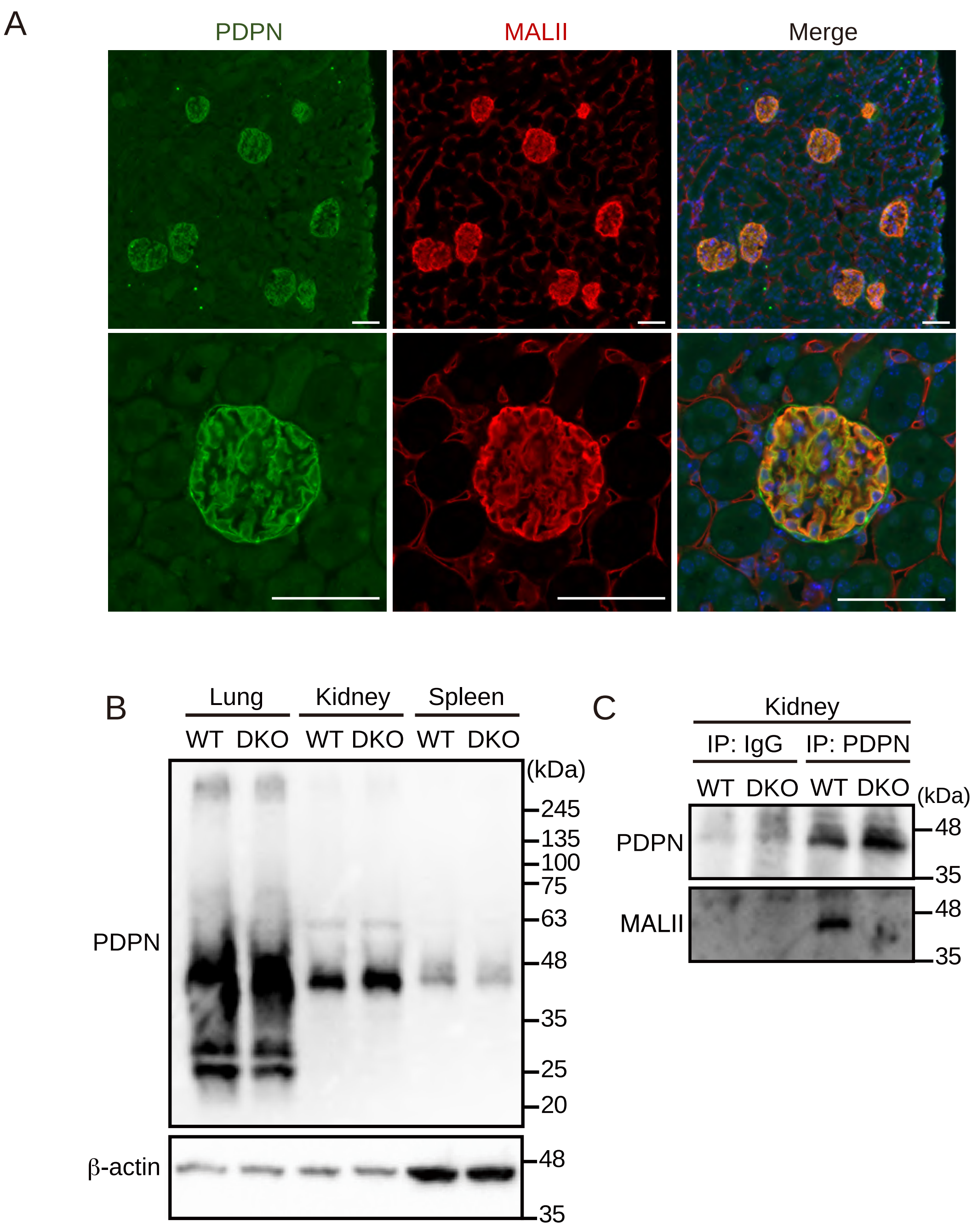

Figure S5. The expression of disialyl-T structures in podoplanin-expressed tissues.
(A) Immunofluorescence staining of podoplanin (PDPN, green) antibody and MALII lectin (red) in wild-type kidney. Lower images show large magnification of a glomeruli. Scale bar; 50 μm. (B) Western blotting of podoplanin using the lung, kidney, and spleen lysates in WT and DKO mice. (C) Immunoprecipitation (IP) of PDPN using the kidney lysates in WT and DKO mice. Control rat IgG was used for detecting the unspecific bands. The IP samples were detected using anti-PDPN antibody and MALII lectin.

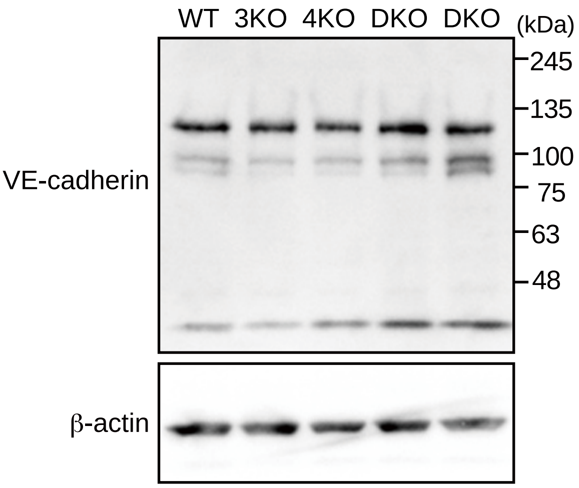

Figure S6. Western blotting of VE-cadherin and β-actin using the lysates of mesenteric lymph nodes in WT, 3KO, 4KO, and DKO.

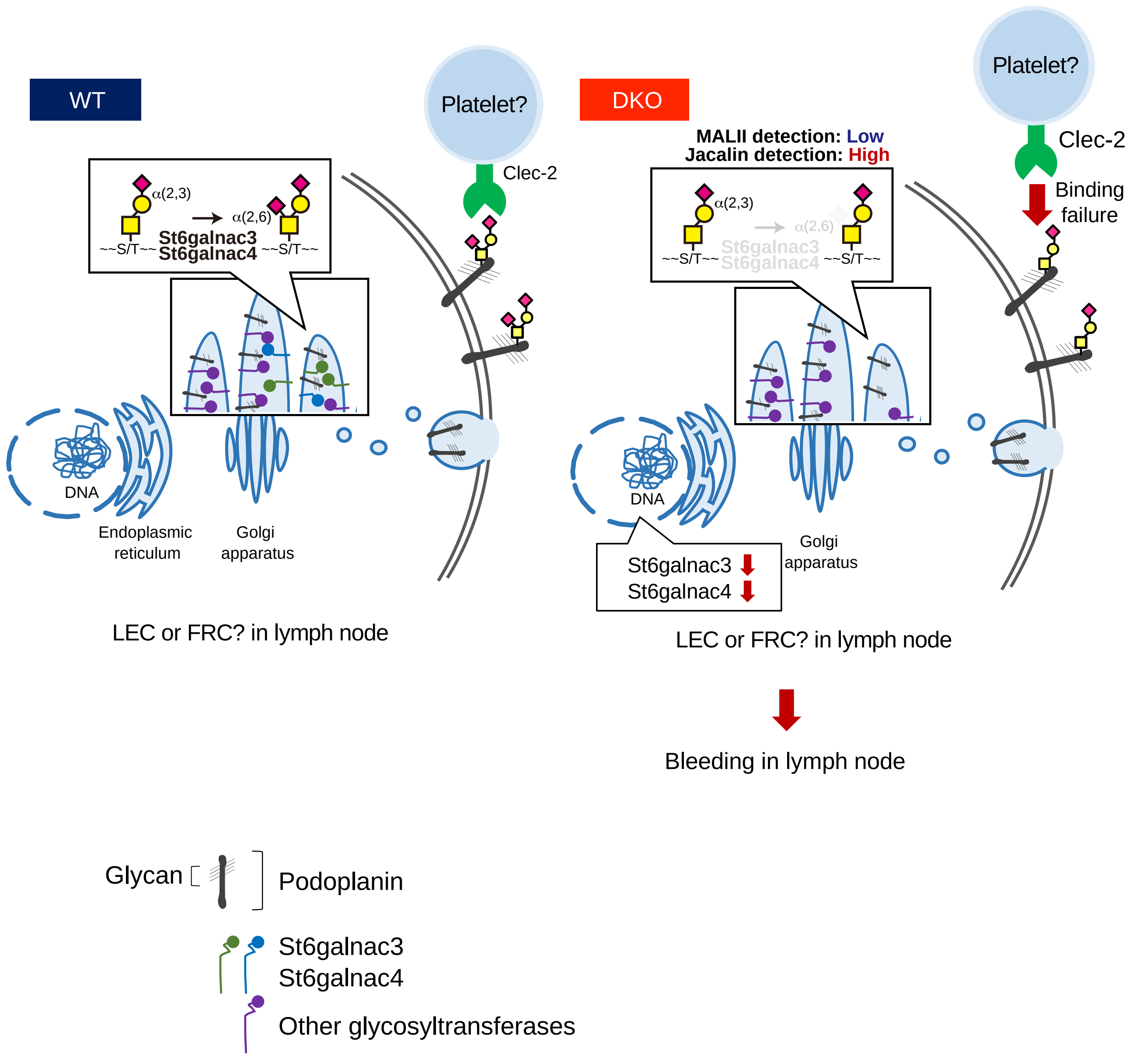

Figure S7. Summary of this study.
In WT (healthy, left panel), one or more disialyl-Ts are modified to podoplanin. disialyl-T-modified podoplanin is recognized by Clec-2.
In DKO mice (right panel), St6galnac3 and 4 are defective and the disialyl-T structures are not modified to podoplanin. This prevents binding of Clec-2 to podoplanin, which may result in bleeding in the lymph nodes. Of note, binding of Clec-2 to podoplanin requires α2,6-linked sialic acid, which is part of the disialyl-T structure.

A

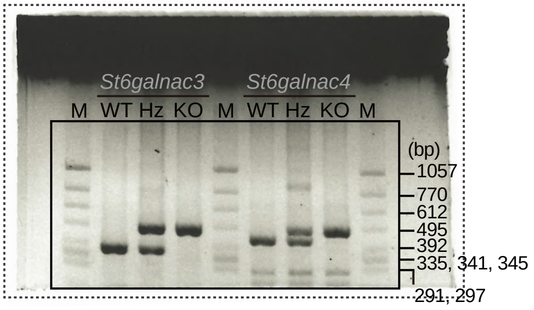

B

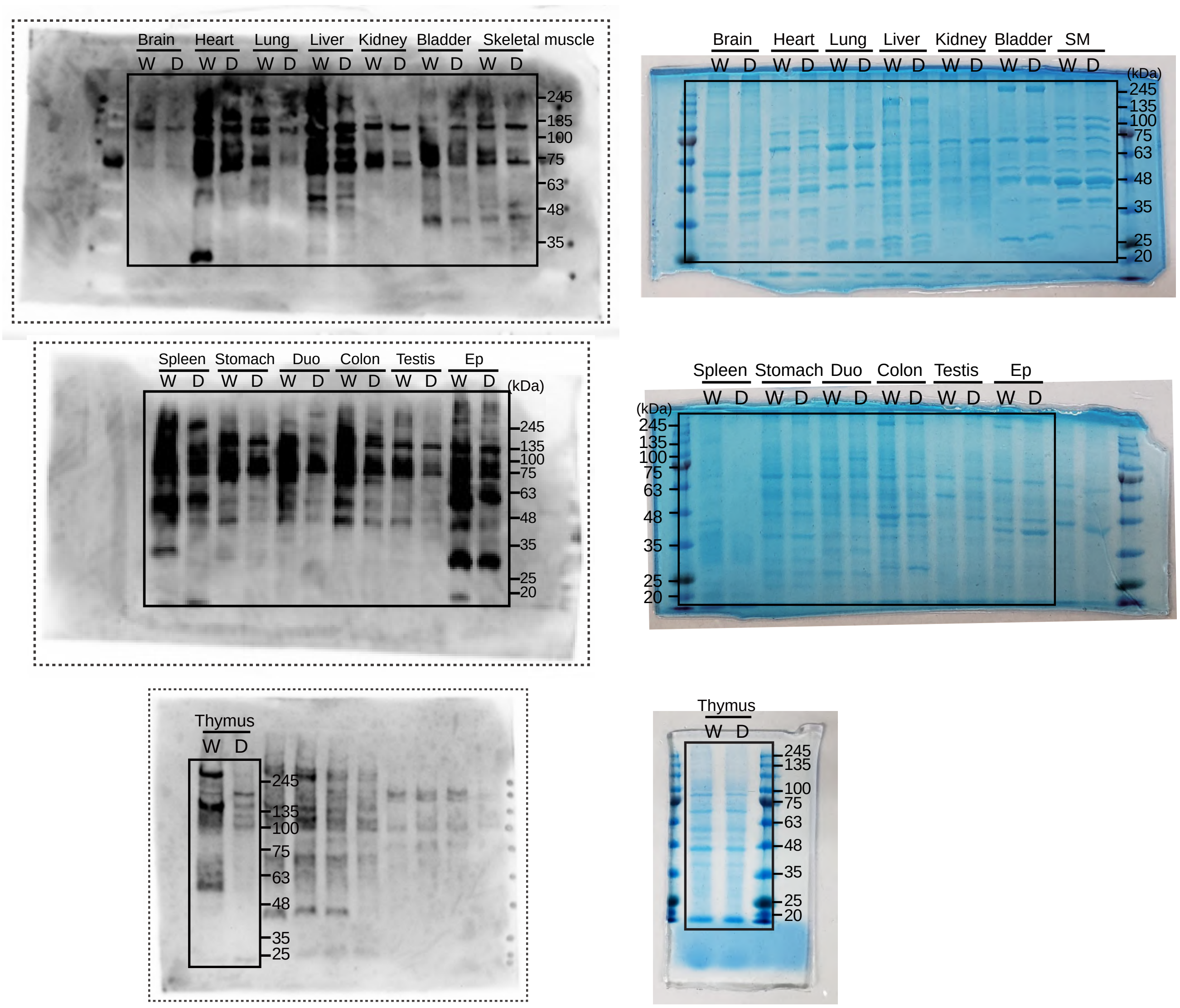

Figure S8. Original images of gel-staining and western blotting.
(A) A original image of Fig. 1C. For the final figure, illustrator was used to crop the square area. The dashed line indicates the extent of an agarose gel. (B) Original images of Fig. 1E. For the final figure, illustrator was used to crop the square area. The dashed line indicates the extent of the membrane.

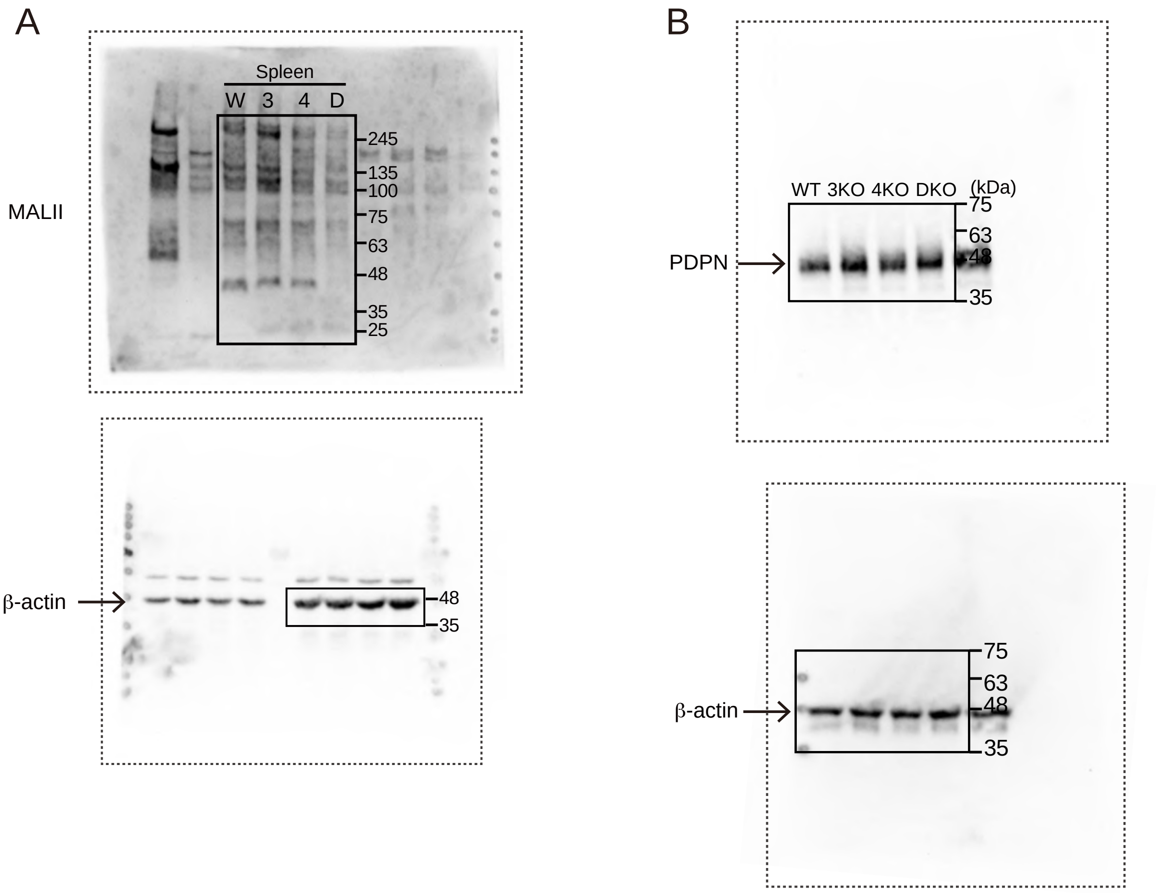

Figure S9. Original images of western blotting.

(A) Original images of Fig. 1H. For the final figure, illustrator was used to crop the square area. The dashed line indicates the extent of the membrane. The membrane was incubated for 15 min in Revitablot Western Blot Stripping Buffer (Rockland) at room temperature to remove bound MALII lectins. After washing three times for 5 min with PBS-T and blocking, the membrane was exposed to anti-β-actin antibody. (B) Original images of Fig. 3B. For the final figure, illustrator was used to crop the square area. The dashed line indicates the extent of the membrane. As mentioned above, the membrane was incubated for 15 min in stripping buffer and the membrane was exposed to anti-β-actin antibody.

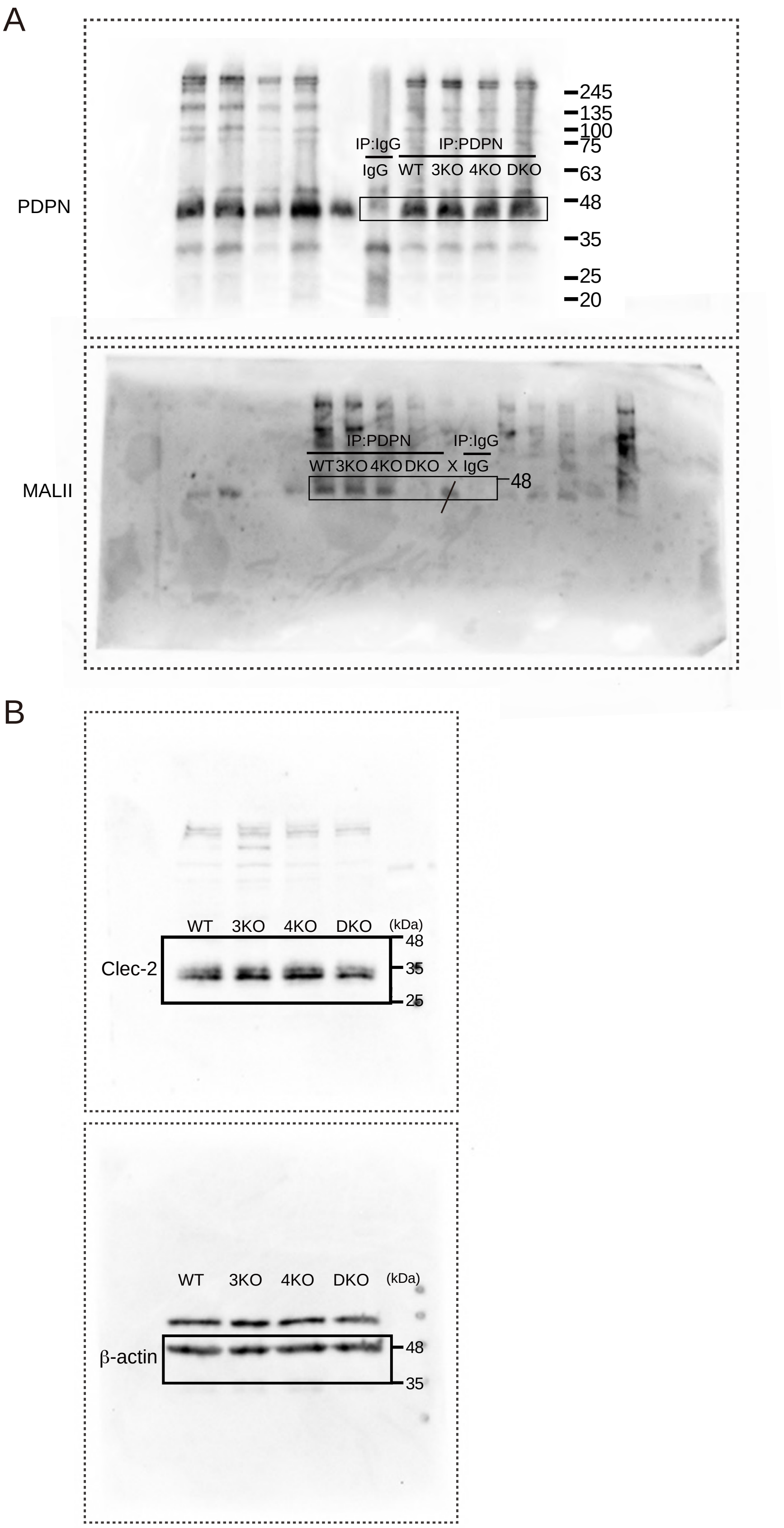

Figure S10. Original images of western blotting.
(A) Original images of Fig. 3D. For the final figure, illustrator was used to crop the square area. The dashed line indicates the extent of the membrane. The band in lane X was removed. (B) Original images of Fig. 3E. For the final figure, illustrator was used to crop the square area. As mentioned above, the membrane was incubated for 15 min in stripping buffer and the membrane was exposed to anti-β-actin antibody. The dashed line indicates the extent of the membrane.

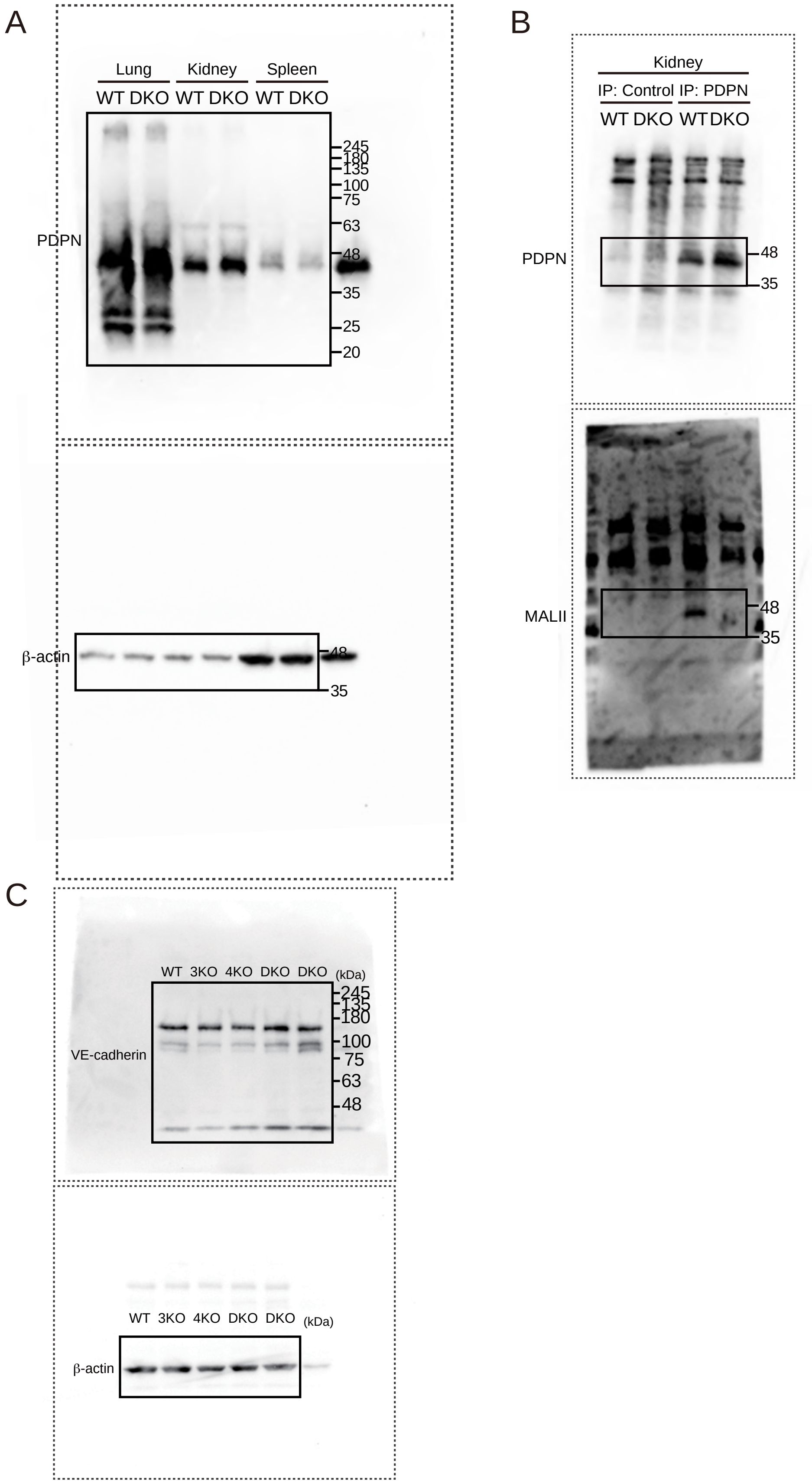

Figure S11. Original images of western blotting.
(A) Original images of Fig. S5B. For the final figure, illustrator was used to crop the square area. The dashed line indicates the extent of the membrane. (B) Original images of Fig. S5C. For the final figure, illustrator was used to crop the square area. The dashed line indicates the extent of the membrane.
(C) Original images of Fig. S6. For the final figure, illustrator was used to crop the square area. As mentioned above, the membrane was incubated for 15 min in stripping buffer and the membrane was exposed to anti-β-actin antibody. The dashed line indicates the extent of the membrane.
